# Designing Multifunctional Antibacterial Hydrogels: A Tri-Pillar Approach Based on Bacteriophages, Hydroxyapatite, and Electrospun Systems

**DOI:** 10.3390/gels12040335

**Published:** 2026-04-17

**Authors:** Jordi Puiggalí

**Affiliations:** Departament de Enginyeria Química, Escola d’Enginyeria de Barcelona Est, Universitat Politècnica de Catalunya, Av. Eduard Maristany 10-14, 08019 Barcelona, Spain; jordi.puiggali@upc.edu

**Keywords:** antibacterial hydrogels, bacteriophages, hydroxyapatite nanoparticles, electrospinning, multifunctional biomaterials, antimicrobial materials, drug delivery systems, biofilm infections, tissue regeneration

## Abstract

The rapid emergence of antibiotic-resistant bacteria represents one of the most critical challenges in modern healthcare and has stimulated intense research into alternative antimicrobial strategies. Antibacterial hydrogels have emerged as versatile biomaterials due to their high water content, tunable physicochemical properties, and ability to function as multifunctional platforms for drug delivery and tissue regeneration. This review analyzes recent advances in antibacterial hydrogel systems through a conceptual framework based on three complementary pillars: biological antibacterial agents, inorganic functional components, and structural material engineering. Biological strategies, particularly bacteriophage-based approaches, provide highly specific antibacterial activity capable of targeting multidrug-resistant pathogens and disrupting bacterial biofilms. Inorganic components such as hydroxyapatite nanoparticles contribute additional functionalities including drug adsorption, modulation of the ionic microenvironment, and osteoconductive behavior relevant for bone-related infections. Structural design strategies based on electrospinning enable the fabrication of fibrous architectures that enhance mechanical stability, regulate therapeutic release, and mimic extracellular matrix organization. The integration of these three pillars within multifunctional hydrogel platforms offers promising opportunities for developing advanced antibacterial biomaterials capable of addressing infection control while supporting tissue regeneration.

## 1. Introduction

The increasing prevalence of antimicrobial resistance (AMR), together with the clinical persistence of biofilm-associated infections, has intensified the search for advanced localized antibacterial strategies capable of overcoming the limitations of conventional antibiotic therapies [1,2,3]. In this context, hydrogels have emerged as highly versatile biomaterials owing to their high water content, structural tunability, and physicochemical resemblance to native tissues [4,5,6,7,8,9,10,11,12,13,14,15].

Among biological antibacterial strategies, bacteriophages have regained considerable attention in response to the global antibiotic resistance crisis. Their capacity to selectively infect bacterial pathogens and amplify at the site of infection offers clear advantages over conventional antimicrobials. However, clinical implementation remains limited by regulatory, manufacturing, and standardization challenges [16,17,18,19,20,21,22]. Recent clinical reports nevertheless highlight the therapeutic potential of phage-based approaches in multidrug-resistant infections [23]. In this context, biomaterial-based delivery systems, particularly hydrogels, are increasingly being explored as platforms to improve phage stability, localization, and antibacterial performance [7,8].

Inorganic bioactive phases also contribute to the development of multifunctional antibacterial biomaterials. Among them, hydroxyapatite has attracted particular interest due to its biocompatibility and its ability to interact with therapeutic agents through adsorption or encapsulation mechanisms [24,25,26]. When incorporated into hydrogels, hydroxyapatite may support controlled delivery while simultaneously promoting tissue integration, especially in bone-related applications.

Structural material design represents an additional key dimension in antibacterial performance. Electrospinning is widely recognized as a versatile and scalable technique for producing micro- and nanofibrous polymeric architectures with controlled morphology, drug incorporation, and release behavior [27,28,29]. Previous studies on electrospun biodegradable polymers loaded with antibacterial agents demonstrated the importance of architecture in modulating antimicrobial efficiency and material functionality, laying the foundation for current hybrid fibrous–hydrogel systems [30]. The integration of electrospun networks within hydrogel matrices therefore represents a logical step toward multifunctional constructs combining hydrated environments with mechanically reinforced scaffolds.

**Figure 1:**
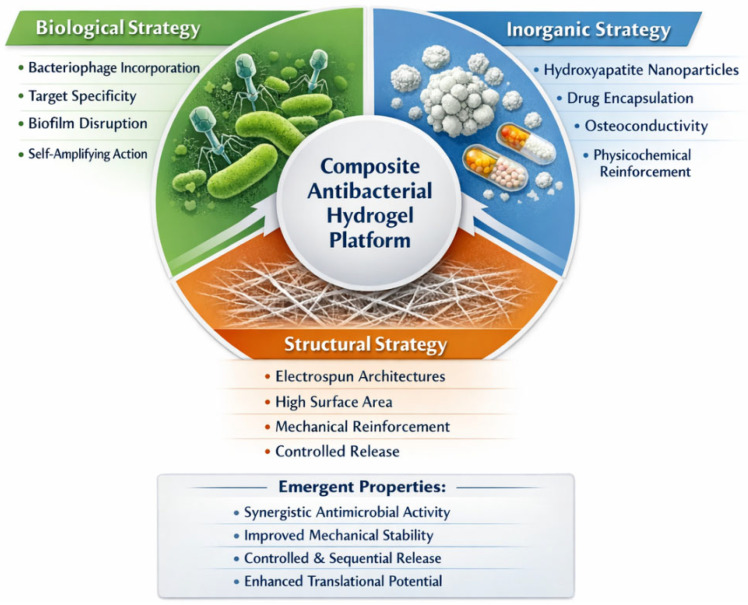
Conceptual framework of multifunctional antibacterial hydrogels based on three complementary design pillars. Biological strategies include bacteriophage incorporation to provide strain-specific antibacterial activity and biofilm disruption. Inorganic components, such as hydroxyapatite nanoparticles, contribute to drug encapsulation, bioactivity, and physicochemical reinforcement. Structural approaches, including electrospun architectures, enhance mechanical performance and enable controlled spatiotemporal release. The integration of these pillars within a composite hydrogel platform results in synergistic and integrated antibacterial systems.

Unlike previous reviews that mainly addressed individual antibacterial strategies or specific material classes, the present work adopts a convergent perspective centered on the combined use of biological, inorganic, and structural design principles within hydrogel platforms. Figure 1 summarizes the tri-pillar conceptual framework, highlighting the interplay between biological, inorganic, and structural strategies in multifunctional antibacterial hydrogels.

This review critically examines antibacterial hydrogels that combine biological, inorganic, and structural functionalities within composite platforms. Particular attention is given to phage-loaded hydrogels, hydroxyapatite-containing formulations, and electrospun–hydrogel hybrids, with emphasis on antimicrobial mechanisms, physicochemical interactions, and controlled release behavior.

Antibacterial hydrogels are currently applied in diverse biomedical contexts, including wound healing, bone regeneration, implant coatings, and localized infection control. In this context, recent advances in dynamic and stimuli-responsive hydrogel systems have further demonstrated the potential of adaptive antibacterial platforms capable of modulating therapeutic activity in response to infection-specific microenvironmental cues, thereby enabling more precise spatiotemporal control over drug release and antibacterial performance [31]. In wound care, hydrogel-based dressings provide a moist environment that supports tissue repair while enabling the sustained release of antimicrobial agents. In bone-related infections, composite hydrogel systems incorporating bioactive inorganic components such as hydroxyapatite have demonstrated potential to simultaneously promote osteointegration and prevent bacterial colonization. These diverse applications highlight the need for integrative design strategies capable of addressing both antibacterial efficacy and tissue regeneration within a single platform. This growing emphasis on integration is consistent with recent work on multifunctional composite biomaterials, which highlights how combining complementary antibacterial mechanisms within a single platform can improve therapeutic efficacy in complex infection scenarios [29].

Several recent reviews have addressed antibacterial hydrogels from specific perspectives, including antimicrobial peptides, nanocomposite systems, and electrospun scaffolds. However, these studies often focus on individual material classes or mechanisms without integrating biological, inorganic, and structural design strategies into a unified framework. In contrast, the present review adopts a tri-pillar approach that emphasizes the synergistic interplay between bacteriophage-based therapies, hydroxyapatite-containing systems, and electrospun architectures, providing a more comprehensive perspective on multifunctional antibacterial hydrogel design.

This review is based on literature retrieved primarily from Web of Science, Scopus, and PubMed, with emphasis on recent studies and reviews addressing antibacterial hydrogels integrating biological, inorganic, and structural design strategies. Priority was given to articles reporting mechanistic insights, representative biomaterial platforms, and translationally relevant performance in wound healing, bone-related infections, and localized antibacterial delivery.

Table 1 compares the three conceptual pillars of multifunctional antibacterial hydrogels in terms of their main functions, advantages, limitations, and contributions to overall system performance. To facilitate comparison between the different design strategies discussed in this review, Table 1 summarizes the main characteristics, advantages, and limitations of the three conceptual pillars underlying multifunctional antibacterial hydrogels.

## 2. Biological Antibacterial Strategies: Bacteriophages

Bacteriophages have re-emerged as promising antibacterial agents in response to the global increase in antimicrobial resistance. Their ability to selectively infect and lyse bacterial cells while amplifying at the site of infection distinguishes them from conventional antibiotics and provides a dynamic mechanism for bacterial control [6,7,8]. Unlike broad-spectrum antimicrobials, bacteriophages exhibit high specificity toward their bacterial hosts, which can minimize disruption of beneficial microbiota and reduce the risk of secondary infections.

### 2.1. Historical and Clinical Perspective of Phage Therapy

The therapeutic use of bacteriophages dates back to the early twentieth century, following their discovery by Twort and d’Hérelle. Despite early clinical applications, phage therapy was largely abandoned in Western medicine after the introduction of antibiotics. However, the rapid emergence of multidrug-resistant bacterial strains has renewed interest in phage-based therapies as alternative or complementary approaches [20].

Recent studies have highlighted both the potential and the limitations of phage therapy. While successful case reports and experimental treatments demonstrate efficacy against resistant infections, large-scale clinical validation remains limited, and regulatory frameworks are still evolving [21,22]. Nevertheless, advances in phage engineering and formulation strategies continue to expand the therapeutic possibilities of phage-based interventions [17,19].

### 2.2. Mechanisms of Antibacterial Action and Biofilm Disruption

Bacteriophages exert their antibacterial activity primarily through infection of bacterial cells followed by replication and lysis. This process not only eliminates individual bacteria but also enables amplification of the therapeutic agent in situ. Importantly, bacteriophages can penetrate and disrupt bacterial biofilms, which are typically resistant to conventional antibiotics due to their dense extracellular matrix and reduced metabolic activity [2,3].

Phage-mediated biofilm disruption involves multiple mechanisms, including enzymatic degradation of extracellular polymeric substances and localized amplification within the biofilm structure. These properties make bacteriophages particularly attractive for treating chronic and biofilm-associated infections, where traditional antimicrobial strategies often fail.

### 2.3. Challenges in Phage Therapy: Resistance, Stability, and Regulation

Despite promising biological attributes, several barriers continue to restrict widespread clinical implementation of phage therapy.

#### 2.3.1. Phage Resistance

Bacteria can develop resistance to phages through receptor modification, CRISPR-Cas systems, or abortive infection mechanisms [17,19]. Although phage resistance may arise rapidly in vitro, it can sometimes be associated with reduced bacterial fitness or restored antibiotic susceptibility. The use of multi-phage cocktails and engineered phage derivatives has been proposed to reduce the likelihood of therapeutic failure [23].

#### 2.3.2. Stability and Storage

Phages are biological entities sensitive to environmental conditions, including temperature, pH, ionic strength, and desiccation [22]. Experimental studies evaluating phage stability in clinically relevant wound care products have demonstrated that matrix composition and storage conditions significantly influence viral infectivity, highlighting the importance of formulation parameters in therapeutic performance [32]. Loss of infectivity during storage or after incorporation into delivery matrices represents a critical limitation. Comprehensive analyses of phage formulation strategies have highlighted the influence of polymer composition, drying methods, and encapsulation approaches on viral stability, emphasizing the need for rational material selection during hydrogel design [33,34]. Encapsulation within polymeric systems may protect phages from inactivation; however, polymerization chemistry and crosslinking conditions must be carefully optimized to avoid compromising viral structure [8].

#### 2.3.3. Regulatory and Manufacturing Constraints

Unlike small-molecule drugs, phage preparations may require adaptation to specific bacterial strains, raising regulatory challenges related to standardization and reproducibility [21,22]. Batch-to-batch variability, genomic characterization, and quality control of biological contaminants complicate large-scale production. These issues reinforce the need for stable, well-characterized delivery systems that can enhance consistency and facilitate regulatory approval pathways.

### 2.4. Integration of Bacteriophages Within Hydrogel Systems

The porous structure of hydrogels facilitates bacteriophage diffusion while preserving a hydrated microenvironment that supports phage stability and activity. In addition, hydrogel networks can be engineered to modulate release kinetics, protect phages from inactivation, and enhance retention at the target site.

Recent studies have demonstrated the successful incorporation of bacteriophages into polymeric hydrogels, preserving their antibacterial activity and enabling controlled release behavior. For example, hydrogel systems based on poly(γ-glutamic acid) have been shown to effectively encapsulate bacteriophages while maintaining their bactericidal activity and allowing for sustained release under physiological conditions [35]. Such systems highlight the potential of hydrogel matrices to act as protective and functional delivery platforms.

**Figure 2:**
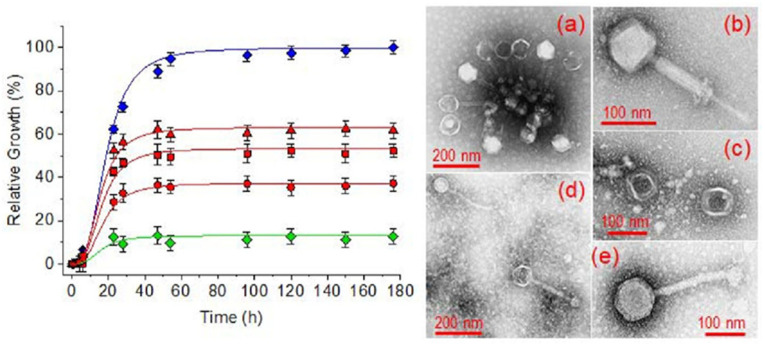
Antibacterial activity of Fersisi bacteriophages against *Staphylococcus aureus* and morphological characterization of the viral particles. The growth curve shows the control bacterial culture without phages (blue) and the relative growth of *S. aureus* in the presence of bacteriophage-loaded electrospun hydrogels with different crosslinking degrees: 100% (circles), 75% (squares), and 50% (triangles), as well as the commercial phage preparation (green). Transmission electron microscopy (TEM) images illustrate the morphology of Fersisi bacteriophages corresponding to different families: (**a**) *Myoviridae*, (**b**) *Siphoviridae*, (**c**) *Leviviridae*, and (**d**,**e**) *Podoviridae*. Reproduced with permission from [35] Kasbiyan et al. (MDPI, CC-BY license).

These concepts are schematically illustrated in Figure 2, which summarizes the incorporation of bacteriophages into hydrogel networks, their stabilization within the polymer matrix, and their subsequent controlled release toward bacterial targets. The ability to maintain phage viability while ensuring localized delivery represents a key advantage of these systems.

Recent advances in phage-based antibacterial hydrogels demonstrate the feasibility of localized bacterial targeting and controlled release strategies [8]. In wound healing contexts, hydrogel dressings combine moisture retention and tissue compatibility with antimicrobial delivery, while early in vivo studies of bacteriophage-loaded hydrogels in burn wound infections showed significant bacterial reduction and improved healing outcomes [36]. In bone-related infections, composite systems integrating hydrogels with bioactive phases such as hydroxyapatite may combine structural support with phage-mediated bacterial control [11,24].

### 2.5. Design Strategies for Phage-Loaded Hydrogels

The successful incorporation of bacteriophages into hydrogel matrices requires careful consideration of both virological and material science parameters. Unlike small-molecule antibiotics, phages are complex biological nanoparticles with defined capsid structures, tail fibers, and enzymatic components that must remain intact to preserve infectivity. Consequently, hydrogel design must balance network formation chemistry, mechanical integrity, and biological compatibility.

#### 2.5.1. Physical Entrapment During Gelation

One of the most common approaches involves direct mixing of phage suspensions with polymer precursors prior to gelation. This strategy has been applied in natural polymer systems (e.g., alginate, gelatin, chitosan) and synthetic hydrogels (e.g., PEG-based networks) [8]. Experimental studies using alginate-based hydrogels have demonstrated that ionic gelation under mild conditions enables efficient phage encapsulation while preserving infectivity and allowing for sustained release profiles compatible with antibacterial activity [37]. Mild crosslinking conditions are essential, as radical polymerization, UV exposure, or reactive crosslinkers may inactivate viral particles.

Entrapment efficiency depends on: (a) polymer concentration, (b) crosslink density, (c) gelation kinetics, and (d) ionic environment.

Higher crosslink densities generally reduce diffusion but may also limit phage mobility and propagation. Therefore, network mesh size must be optimized to allow for viral diffusion toward bacterial targets while preventing premature loss from the matrix.

#### 2.5.2. Post-Gel Loading and Diffusion-Based Incorporation

Alternatively, preformed hydrogels can be loaded by soaking in concentrated phage suspensions. Diffusion-driven loading minimizes exposure to reactive chemistry but may result in surface-localized distribution and burst release. Strategies to improve retention include electrostatic interactions between negatively charged phage capsids and cationic polymer networks.

Recent studies have explored affinity-based incorporation through surface-functionalized hydrogels, enabling reversible binding and prolonged release [8,17].

#### 2.5.3. Microencapsulation and Multi-Compartment Systems

Advanced strategies include microgel encapsulation, layer-by-layer assemblies, and core–shell architectures. Encapsulating phages within secondary microcarriers prior to hydrogel incorporation can provide: (a) additional protection against environmental stress, (b) hierarchical release profiles, and (c) separation of incompatible components.

Such multi-compartment systems may be particularly useful when combining phages with antibiotics, peptides, or inorganic nanoparticles within a single platform.

#### 2.5.4. Stimuli-Responsive and Infection-Triggered Release

Smart hydrogel systems capable of responding to environmental cues offer promising solutions to the dynamic nature of bacterial infections. pH-responsive, enzyme-sensitive, or temperature-sensitive networks may enable on-demand phage release under infection-specific conditions.

For example: acidic microenvironments in infected wounds, bacterial enzyme secretion and inflammatory mediator presence.

Stimuli-responsive systems align closely with current advances in multifunctional hydrogel engineering [29] and with recent developments in phage-based hydrogel platforms [8].

#### 2.5.5. Integration with Structural and Inorganic Components

Hybrid constructs combining phage-loaded hydrogels with electrospun fibrous mats or hydroxyapatite nanoparticles represent an emerging direction. Electrospun layers may serve as mechanical reinforcement or controlled release interfaces [27,30], while hydroxyapatite can provide drug co-loading capacity and osteoconductivity in bone infection scenarios [11,24].

Such composite designs embody the tri-pillar framework proposed in this review, integrating biological specificity, inorganic functionality, and structural optimization within a unified antibacterial platform.

**Figure 3:**
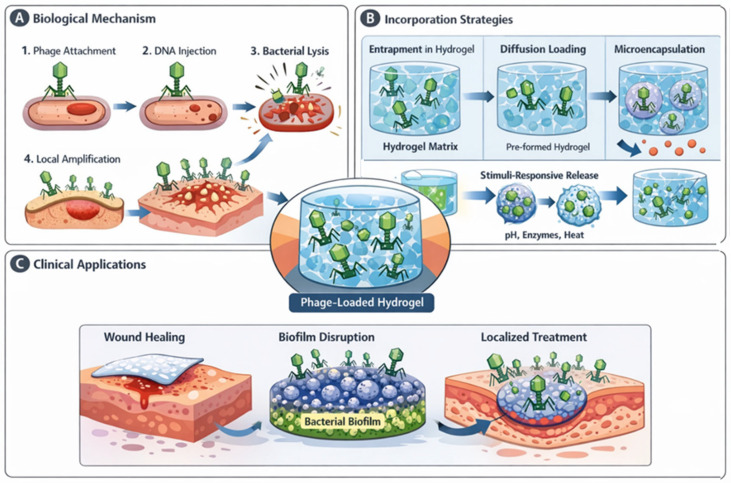
Design principles and therapeutic mechanisms of bacteriophage-loaded hydrogels. (**A**) Biological mechanism of lytic bacteriophages, including bacterial attachment, DNA injection, host cell lysis, and local viral amplification. (**B**) Representative strategies for phage incorporation into hydrogel matrices, including direct entrapment during gelation, diffusion-based loading into preformed networks, microencapsulation approaches, and stimuli-responsive release systems triggered by environmental cues such as pH, enzymes, or temperature. (**C**) Clinical applications of phage-loaded hydrogels, illustrating wound healing support, biofilm disruption, and localized antibacterial treatment. The schematic highlights the role of hydrogels as protective reservoirs that preserve phage viability while enabling controlled and site-specific antimicrobial action.

The main strategies for bacteriophage incorporation into hydrogel systems, together with the associated stabilization challenges and release behaviors that determine antibacterial efficacy, are schematically summarized in Figure 3.

A comparative overview of the main strategies for incorporating bacteriophages into hydrogel systems, together with their key advantages and limitations, is presented in Table 2. This table emphasizes the relationship between material parameters (e.g., crosslink density, mesh size, polymer chemistry) and biological performance (e.g., phage stability, release kinetics, and antibacterial efficacy).

Despite the diversity of incorporation strategies, each approach presents specific advantages and limitations. Physical entrapment during gelation offers simplicity and high encapsulation efficiency but may expose phages to potentially damaging crosslinking conditions. Post-gel loading strategies preserve phage integrity but often result in non-uniform distribution and rapid initial release. Advanced approaches such as microencapsulation and multi-compartment systems provide improved protection and controlled release profiles, although they introduce additional complexity in fabrication and scalability. Therefore, the selection of an appropriate incorporation strategy must balance phage stability, release kinetics, and practical considerations related to manufacturing and clinical translation.

## 3. Inorganic Pillar: Hydroxyapatite-Based Functionalization in Antibacterial Hydrogels

While biological strategies provide specificity and self-amplifying antibacterial mechanisms, the incorporation of inorganic components introduces an additional level of physicochemical functionality to hydrogel-based systems. Among the various bioactive inorganic phases explored in biomedical materials, hydroxyapatite (HA) occupies a particularly prominent position due to its structural similarity to the mineral component of bone, its intrinsic biocompatibility, and its versatile surface chemistry [9,10,11]. Within antibacterial hydrogel platforms, hydroxyapatite does not primarily function as a direct bactericidal agent but rather as a multifunctional inorganic phase capable of modulating mechanical properties, drug loading capacity, interfacial interactions, and tissue integration.

The integration of hydroxyapatite into hydrogel matrices enables the development of composite systems that combine the hydrated, diffusion-permissive environment of polymers with the rigidity and bioactivity of inorganic nanophases. This combination is especially relevant in the context of infected bone defects, chronic wounds involving mineralized tissues, and implant-associated infections, where both antimicrobial control and tissue regeneration are required simultaneously. In such scenarios, hydroxyapatite-containing hydrogels may serve as dual-function platforms that provide structural cues for osteoconduction while acting as reservoirs for antibacterial agents.

**Figure 4:**
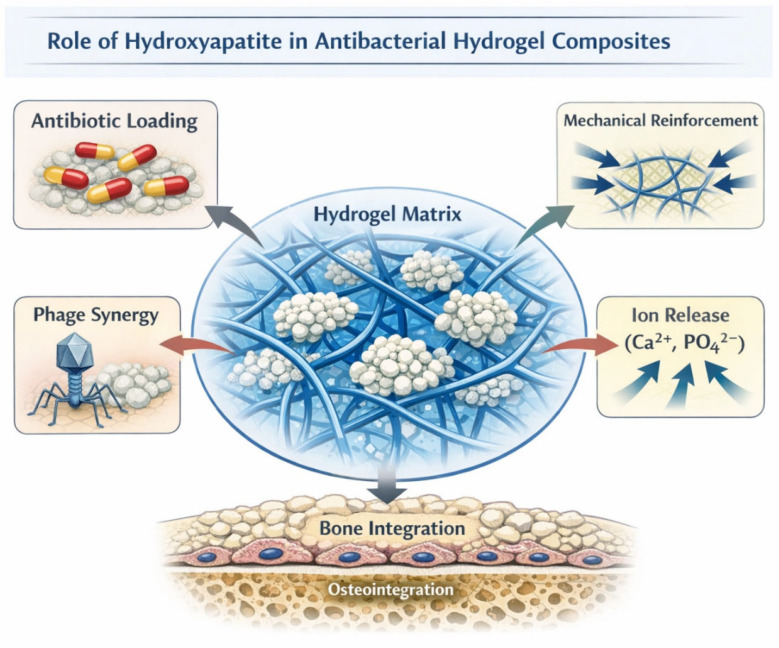
Multifunctional role of hydroxyapatite in antibacterial hydrogel composites. Schematic representation of hydroxyapatite (HA) nanoparticles dispersed within a hydrogel matrix and their contribution to composite performance. HA particles act as adsorption sites for therapeutic agents (e.g., antibiotics), modulate mechanical properties through inorganic reinforcement, and influence ion release (Ca^2+^ and PO_4_^3−^) under physiological or mildly acidic conditions. The composite system may further support bone integration in infection-related defects by combining structural bioactivity with localized antibacterial functionality. The figure highlights the role of HA as a physicochemically active inorganic phase within multifunctional hydrogel platforms.

From a material science perspective, the contribution of hydroxyapatite extends beyond simple filler reinforcement. Nanoscale HA particles exhibit high specific surface area, tunable crystallinity, and modifiable surface charge, allowing for strong interactions with both polymer networks and therapeutic molecules [24,25,26]. These characteristics support controlled adsorption–desorption processes, influence hydrogel swelling behavior, and may alter degradation kinetics. Consequently, the inorganic pillar of antibacterial hydrogels should be understood not merely as an additive component but as a functional phase capable of redefining the physicochemical landscape of the composite system. The physicochemical interactions governing drug adsorption onto hydroxyapatite surfaces are schematically summarized in Figure 4, highlighting the role of surface charge, functional groups, and environmental conditions in controlling adsorption–desorption behavior.

In this section, the physicochemical basis of hydroxyapatite in hydrogel composites is first examined, followed by its role as a drug reservoir and structural modulator within antibacterial platforms.

### 3.1. Physicochemical Basis of Hydroxyapatite in Biomaterials

Hydroxyapatite (Ca_10_(PO_4_)_6_(OH)_2_) is a calcium phosphate mineral characterized by a hexagonal crystal structure and a Ca/P molar ratio of 1.67 in its stoichiometric form. Its crystallographic arrangement enables substitutional flexibility, allowing for partial replacement of calcium, phosphate, or hydroxyl ions with alternative ionic species. This compositional tunability contributes to its widespread use in biomedical applications, as ionic substitutions can modulate solubility, surface charge, and biological response [9,10].

At the nanoscale, hydroxyapatite particles typically present rod-like or plate-like morphologies, with dimensions ranging from tens to hundreds of nanometers. Reduction in particle size significantly increases specific surface area, thereby enhancing adsorption capacity and interfacial reactivity. Surface hydroxyl and phosphate groups participate in hydrogen bonding, electrostatic interactions, and coordination with functional groups present in polymer chains. When dispersed within hydrogel matrices, these interactions may lead to: (a) increased crosslinking density through physical or ionic interactions, (b) modification in network architecture, (c) alteration in swelling equilibrium, and (d) enhancement in mechanical stiffness.

Such effects are particularly pronounced in nanocomposite hydrogels, where homogeneous dispersion of HA nanoparticles results in improved load transfer and resistance to deformation [11,24].

Another fundamental physicochemical property of hydroxyapatite is its pH-dependent solubility. Under slightly acidic conditions—often encountered in infected or inflamed tissues—HA dissolution may increase, leading to localized release of calcium and phosphate ions. Although not inherently bactericidal, these ionic fluxes can influence cellular behavior, mineralization processes, and potentially local microenvironmental conditions. The buffering capacity associated with calcium phosphate systems may also contribute to stabilization of local pH within composite materials.

**Figure 5:**
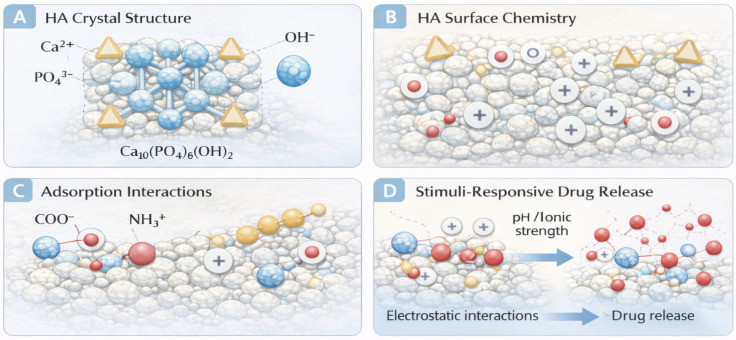
Surface chemistry and adsorption mechanisms of hydroxyapatite. (**A**) Simplified representation of the hydroxyapatite crystal structure showing Ca^2+^ ions, PO_4_^3−^ tetrahedra, and hydroxyl groups (OH^−^) arranged in the apatite lattice (Ca_10_(PO_4_)_6_(OH)_2_). (**B**) Surface chemistry of hydroxyapatite nanoparticles highlighting surface charge distribution and exposed functional groups. (**C**) Representative adsorption interactions between hydroxyapatite surfaces and drug molecules containing carboxylate (–COO^−^) or protonated amine (–NH_3_^+^) functionalities. (**D**) Schematic representation of stimuli-responsive drug release from hydroxyapatite surfaces, where changes in the local environment, such as pH or ionic strength, weaken electrostatic interactions and trigger the controlled desorption of adsorbed molecules.

The interaction between hydroxyapatite and therapeutic agents is governed by surface charge distribution and the presence of active adsorption sites. The main structural and surface-chemistry features governing adsorption and release phenomena in hydroxyapatite are schematically summarized in Figure 5. Antibiotics bearing carboxylate or amine groups can bind through electrostatic interactions or chelation with calcium ions, enabling reversible adsorption and sustained release profiles. The strength of these interactions depends on particle crystallinity, surface functionalization, and ionic environment. Consequently, the design of HA-containing hydrogels requires careful consideration of: (a) particle size distribution, (b) degree of crystallinity, (c) surface modification strategies, and (d) polymer compatibility.

When incorporated into hydrogels, hydroxyapatite may also influence diffusion pathways. The presence of dispersed inorganic domains can create tortuous transport routes, thereby reducing burst release of encapsulated agents and contributing to prolonged antibacterial activity. Furthermore, interfacial interactions between HA particles and polymer chains may stabilize the composite against rapid degradation.

Importantly, hydroxyapatite’s biocompatibility and osteoconductivity provide a critical advantage in applications involving mineralized tissues. In bone-related infections, the use of HA-containing hydrogels may facilitate simultaneous antimicrobial therapy and regenerative support. Unlike purely polymeric systems, these composites offer a structural and biochemical environment more closely aligned with native bone matrix, potentially enhancing integration and long-term functionality.

Taken together, the physicochemical characteristics of hydroxyapatite—crystallographic tunability, surface reactivity, adsorption capacity, ionic responsiveness, and mechanical reinforcement potential—justify its inclusion as a key inorganic component within multifunctional antibacterial hydrogel platforms.

### 3.2. Hydroxyapatite as a Drug Reservoir in Hydrogel Systems

One of the most significant contributions of hydroxyapatite within antibacterial hydrogel composites lies in its function as an inorganic drug reservoir. Unlike purely polymeric systems in which therapeutic agents are primarily retained through physical entrapment within the hydrogel mesh, hydroxyapatite provides additional adsorption sites that enable reversible binding and controlled release of bioactive molecules. This dual retention mechanism—polymeric confinement combined with inorganic adsorption—creates hierarchical delivery platforms with enhanced control over drug kinetics. Hydroxyapatite nanoparticles have also been incorporated into polymeric systems as multifunctional platforms for drug delivery and biomedical applications, demonstrating promising biocompatibility and therapeutic potential [24].

Recent studies have further expanded the role of hydroxyapatite-based systems beyond their classical use as passive osteoconductive materials, emphasizing their capacity to act as active platforms for therapeutic delivery and microenvironment modulation. In particular, the tunable surface chemistry and ion substitution capability of hydroxyapatite nanoparticles have been exploited to regulate drug adsorption, control release kinetics, and influence local biological responses, including antibacterial activity and cellular behavior [38]. In this context, increasing evidence indicates that ion-substituted calcium phosphate materials can exhibit intrinsic antibacterial activity through ion release and surface-mediated interactions, although the magnitude and reproducibility of these effects strongly depend on the type and concentration of substituted ions as well as the specific material formulation [39]. Furthermore, it is increasingly recognized that calcium phosphate nanoparticles may also exhibit intrinsic antibacterial effects through ion-mediated mechanisms and surface interactions with bacterial membranes, further expanding their role beyond passive carriers in antibacterial biomaterials [40]. However, despite these promising features, several limitations remain associated with the use of hydroxyapatite nanoparticles in antibacterial systems. These include potential aggregation phenomena, limited intrinsic antibacterial activity in the absence of functionalization, and challenges in achieving controlled and reproducible ion release profiles under physiological conditions. Moreover, the incorporation of hydroxyapatite into complex composite systems may alter mechanical properties and degradation behavior, requiring careful optimization to balance structural stability with biological performance [41].

#### 3.2.1. Mechanisms of Drug Adsorption onto Hydroxyapatite

Drug adsorption onto hydroxyapatite is governed by a combination of electrostatic interactions, ion exchange, hydrogen bonding, and coordination chemistry [9,10,42]. The surface of HA nanoparticles presents exposed calcium ions (Ca^2+^), phosphate groups (PO_4_^3−^), and hydroxyl groups, all of which may participate in molecular interactions [9,42].

Antibiotics containing carboxylate groups (–COO^−^) can chelate surface calcium ions through coordination bonding, whereas molecules bearing protonated amine groups (–NH_3_^+^) may interact electrostatically with negatively charged phosphate sites [43,44]. In some cases, adsorption involves multi-point interactions, increasing binding affinity and slowing desorption kinetics.

The extent of adsorption depends on: (a) surface area and particle size (nano- vs. micro-HA), (b) degree of crystallinity, (c) surface functionalization, (d) solution pH and ionic strength, and (e) drug molecular structure and ionization state.

Under physiological conditions, HA typically exhibits a slightly negative surface charge, favoring adsorption of cationic species. However, local environmental changes—such as the mildly acidic conditions observed in infected tissues—may alter surface charge distribution and solubility, thereby influencing release behavior [42].

#### 3.2.2. Thermodynamics and Kinetics of Adsorption–Desorption

Adsorption processes on hydroxyapatite surfaces frequently follow Langmuir- or Freundlich-type isotherms, depending on surface heterogeneity and interaction strength [38,40]. In nanostructured HA systems, heterogeneous binding sites often lead to non-ideal adsorption profiles and multi-phase release kinetics.

From a kinetic perspective, drug release from HA-containing hydrogels may involve: (a) desorption from HA surface, (b) diffusion through the hydrogel network, and (c) possible hydrogel degradation.

This multi-step mechanism contrasts with simple Fickian diffusion typically observed in drug-loaded hydrogels lacking inorganic phases. As a result, HA incorporation may reduce initial burst release and promote sustained release over extended periods [11,24,26,44].

Importantly, adsorption strength must be carefully balanced: excessively strong binding may impair therapeutic availability, whereas weak interactions may result in premature release. Rational design therefore requires tuning HA surface properties—particle size, crystallinity, and functionalization—to achieve optimal desorption rates [43,44].

#### 3.2.3. Influence of Hydrogel–HA Interactions on Release Profiles

The presence of hydroxyapatite modifies hydrogel architecture in several ways that indirectly affect drug release: (a) increased tortuosity of diffusion pathways, (b) reduced effective mesh size, (c) altered swelling equilibrium, and (d) modified degradation kinetics [11,24,29].

In composite systems, drug molecules may be simultaneously retained within the polymeric network and adsorbed onto HA surfaces. This dual retention mechanism generates a two-stage release profile: Initial diffusion-controlled release from the polymer phase, and secondary release governed by surface desorption from HA.

Such hierarchical kinetics have been reported in HA–polymer composite systems designed for sustained antibiotic delivery [26,44,45]. Furthermore, HA nanoparticles may function as localized high-concentration micro-reservoirs, maintaining therapeutic gradients even after partial hydrogel degradation.

#### 3.2.4. Applications in Infected Bone Defects

In osteomyelitis and implant-associated infections, local antibiotic delivery is often preferred over systemic administration in order to minimize toxicity and achieve high local concentrations. HA-containing hydrogels offer several advantages in this context: (a) osteoconductive scaffold supporting bone regeneration, (b) localized antibiotic reservoir reducing systemic exposure, and (c) potential reduction in bacterial colonization on implant surfaces [10,11,46].

Unlike polymethylmethacrylate (PMMA)-based antibiotic carriers, HA–hydrogel composites are typically biodegradable and may support tissue integration. Additionally, gradual HA dissolution under mildly acidic inflammatory conditions may contribute to sustained ion release and remodeling support [42].

In this scenario, the inorganic pillar becomes synergistic with the biological and structural pillars described earlier: HA enables efficient antibiotic retention, hydrogels provide hydrated diffusion environments, and electrospun architectures may enhance mechanical stability and spatial control.

#### 3.2.5. Limitations and Design Considerations

Despite its advantages, hydroxyapatite-based drug delivery systems present several challenges: (a) batch-to-batch variability in nanoparticle size and crystallinity, (b) particle aggregation within polymer matrices, (c) limited loading efficiency for hydrophobic drugs, and (d) risk of excessively slow release due to strong adsorption [43,44].

Surface modification strategies—including ionic substitution, polymer grafting, and chemical functionalization—have been explored to tailor adsorption behavior and improve dispersion within hydrogels [36,44]. Careful optimization of HA content is essential, as excessive inorganic loading may compromise hydrogel elasticity, injectability, or structural homogeneity.

Hydroxyapatite should therefore be regarded not as a passive filler but as a tunable inorganic reservoir whose physicochemical parameters critically determine antibacterial performance and therapeutic durability.

Representative examples of hydroxyapatite-based hydrogel systems developed for antibacterial and bone-related applications are comparatively summarized in Table 3. The table highlights hydroxyapatite characteristics, hydrogel type, incorporated antibacterial agents, intended applications, and reported key outcomes.

### 3.3. Indirect Antibacterial Contributions of Hydroxyapatite

Hydroxyapatite is not typically considered a potent intrinsic antibacterial agent in the same sense as silver nanoparticles, cationic polymers, or antimicrobial peptides. Nevertheless, within antibacterial hydrogel platforms it can contribute to infection control through a series of indirect, yet mechanistically relevant, physicochemical and biological effects. These contributions arise from (i) modulation of the local microenvironment, (ii) altered interfacial interactions with bacteria and host tissue, (iii) enhancement in carrier performance for antibacterial payloads, and (iv) facilitation of tissue integration, particularly in mineralized settings where poor integration can promote persistent infection niches. In this respect, hydroxyapatite acts less as an antimicrobial “active” and more as a multifunctional inorganic phase that shifts the balance of conditions governing bacterial persistence versus host-driven regeneration [9,10,11,42,43,44].

#### 3.3.1. Microenvironmental Modulation and Ionic Effects

A defining feature of calcium phosphate phases is their pH-dependent solubility. In infected or inflamed tissues, local acidity may develop due to bacterial metabolism and host inflammatory responses. Under such conditions, HA dissolution can increase, resulting in localized release of Ca^2+^ and phosphate species [9,10,42]. Although these ions are not antibacterial per se, they can influence multiple processes relevant to infection resolution. Calcium ions modulate cell adhesion and signaling pathways in osteogenic and immune cells, while phosphate availability contributes to mineralization dynamics and can impact local buffering behavior. By contributing to a microenvironment more supportive of tissue repair and mineral deposition, HA-containing composites may reduce dead space and microcavities where bacteria can persist, indirectly lowering the probability of chronic infection recurrence.

Additionally, calcium phosphate phases can exhibit some buffering capacity. Even modest stabilization of local pH may influence both bacterial growth dynamics and the activity profile of pH-sensitive antibacterial agents. This buffering effect is particularly relevant when HA is integrated into hydrogel systems designed for controlled release, where release behavior and agent stability can be pH dependent [11,24,25,26,42].

#### 3.3.2. Interfacial Interactions with Bacteria and Biofilms

Bacterial adhesion is strongly governed by interfacial physicochemistry (surface charge, roughness, hydrophilicity, and the availability of adsorption sites for proteins that mediate bacterial attachment). Hydroxyapatite surfaces can adsorb proteins from biological fluids, forming conditioning layers that subsequently influence bacterial colonization. When HA nanoparticles are dispersed within hydrogel matrices, they may alter local interfacial heterogeneity and surface energy at the hydrogel–tissue and hydrogel–fluid interfaces. While this does not guarantee reduced bacterial adhesion, it can influence biofilm organization and stability at the material interface, particularly when coupled with therapeutic payloads that suppress bacterial growth [10,11,42,43,44].

Importantly, the ability of HA to act as an adsorption phase for antibacterial molecules is itself an indirect antibacterial mechanism: by retaining antibiotics (or other antimicrobial compounds) near the interface where bacteria attempt to colonize, HA can help maintain locally elevated concentrations that suppress early biofilm establishment. This is especially relevant in composite systems where the polymer network alone might permit rapid diffusion and clearance of the agent from the implantation site [11,24,25,26,39].

#### 3.3.3. Enhancement in Antibacterial Payload Performance

From the standpoint of antibacterial hydrogel engineering, one of the most consequential “indirect” effects of HA is kinetic: altering release profiles in ways that improve pharmacodynamic relevance. Pure hydrogels often exhibit an initial burst release, followed by a rapid decline in concentration. In chronic infections or implant-associated scenarios, this kinetic profile can be suboptimal. HA introduces surface-mediated adsorption–desorption steps that can reduce burst release and prolong delivery, thereby improving time-above-MIC (for antibiotics) or extending exposure windows required for effective bacterial clearance [11,24,25,26,42,43,44].

This effect becomes more significant when the composite design is optimized to exploit hierarchical retention: partial drug retention in the polymer mesh combined with stronger, reversible binding on HA surfaces. In practice, the antibacterial “contribution” of HA is thus implemented through improved delivery performance—enhancing stability, prolonging exposure, and enabling more predictable local dosing [11,43,44,45]. Moreover, by influencing diffusion tortuosity and swelling behavior, HA can further modulate transport phenomena in ways that support sustained antibacterial function without excessive total drug loading.

#### 3.3.4. Synergy with Regenerative Performance as Infection Control Strategy

A critical, sometimes underappreciated, link between regeneration and infection control is the role of tissue integration in eliminating protected bacterial reservoirs. In bone-related infections, dead space and poorly integrated biomaterials can become persistent foci for bacteria. Hydroxyapatite’s osteoconductive properties may reduce these vulnerabilities by promoting tissue ingrowth and improving interfacial sealing at bone–material boundaries [9,10,11,24,42,45,46]. In this sense, accelerated or improved integration is itself an anti-infective mechanism: it reduces niches, improves vascularization and immune access, and supports remodeling processes that can help eradicate residual bacterial populations.

This conceptual coupling—antibacterial delivery plus osteoconduction—forms a major rationale for HA-containing hydrogel systems in infected bone defects. The composite is not merely a “drug depot”; it is a bioactive environment designed to both suppress bacterial burden and promote structural repair, thereby reducing the probability of chronic infection relapse [10,11,45,46].

#### 3.3.5. Design Implications and Limitations

While these indirect antibacterial contributions are compelling, it is important to remain critical. HA incorporation does not automatically reduce bacterial adhesion, nor does ion release ensure antibacterial activity. In some contexts, increased surface roughness and heterogeneity can even facilitate bacterial attachment if not paired with effective antimicrobial payloads. Furthermore, excessive HA loading can compromise hydrogel injectability, elasticity, and homogeneity, potentially creating brittle domains or aggregation that weakens both mechanical and transport properties [11,24,25,26,43,44]. Therefore, HA must be integrated through rational composite design, balancing mechanical reinforcement, adsorption behavior, and biological performance.

In summary, hydroxyapatite contributes to antibacterial hydrogel function primarily through indirect pathways: microenvironmental modulation, interfacial effects, and—most prominently—enhanced local delivery and retention of antibacterial agents coupled with improved tissue integration. These combined effects justify its position as a central inorganic component in multifunctional hydrogel platforms aimed at complex infection scenarios, particularly in bone-related applications.

### 3.4. Ionic Release and Antibacterial Effects

Although hydroxyapatite is generally considered a bioinert or bioactive mineral rather than a strongly antibacterial material, the release of ionic species from calcium phosphate phases can influence microbial behavior and contribute indirectly to antibacterial performance. Ionic dissolution processes, particularly under physiologically relevant or mildly acidic conditions, may affect bacterial adhesion, membrane stability, and biofilm formation. In hydrogel–hydroxyapatite composite systems, these ionic effects can operate in parallel with drug delivery mechanisms and surface-mediated interactions, creating a multifactorial antibacterial environment.

#### 3.4.1. Dissolution of Calcium and Phosphate Ions

Hydroxyapatite exhibits limited but measurable solubility in aqueous environments. The dissolution process releases calcium (Ca^2+^) and phosphate (PO_4_^3−^) ions into the surrounding medium, particularly when local pH decreases as a result of inflammation or bacterial metabolism. This ionic exchange is influenced by factors such as crystallinity, particle size, and substitution within the HA lattice [47].

Although Ca^2+^ and phosphate ions are not inherently bactericidal, elevated ionic concentrations can modify osmotic balance and interfere with bacterial metabolic processes. Moreover, fluctuations in ionic composition may affect the physicochemical conditions required for bacterial adhesion and biofilm maturation. In mineralized tissues, the release of these ions can also promote mineralization processes that gradually replace infected or damaged tissue with newly formed bone, thereby reducing bacterial colonization niches [9,10,47].

#### 3.4.2. Ionic Effects on Bacterial Adhesion and Biofilm Development

Bacterial colonization of biomaterial surfaces is strongly governed by electrostatic and physicochemical interactions. The release of calcium ions from hydroxyapatite may alter surface charge conditions and influence bacterial adhesion dynamics. Several studies have shown that calcium-rich environments can modify the structure of extracellular polymeric substances within biofilms, potentially affecting biofilm stability and architecture [48].

In addition, local ionic gradients generated by HA dissolution may interfere with early bacterial attachment events by modifying protein adsorption layers and surface hydration properties. Although these effects are typically moderate compared with those produced by classical antimicrobial agents, they can contribute to a cumulative antibacterial response when combined with drug-loaded or biologically active hydrogel systems.

#### 3.4.3. Ion-Substituted Hydroxyapatites and Enhanced Antibacterial Properties

A significant body of research has explored the incorporation of antibacterial ions into the hydroxyapatite crystal lattice. Substitution of calcium or phosphate sites with ions such as silver (Ag^+^), zinc (Zn^2+^), copper (Cu^2+^), or strontium (Sr^2+^) can impart enhanced antibacterial activity while maintaining the structural properties of the HA framework [41,49,50].

Silver-doped hydroxyapatite is among the most widely investigated systems due to the well-known antimicrobial activity of Ag^+^ ions. These ions can disrupt bacterial membranes, interfere with enzymatic activity, and generate reactive oxygen species that damage microbial cells. Similarly, zinc-substituted HA has demonstrated bacteriostatic effects against several Gram-positive and Gram-negative pathogens while simultaneously supporting osteogenic processes [41,50].

When incorporated into hydrogel matrices, ion-substituted HA nanoparticles can function as localized sources of antibacterial ions. The hydrogel network may regulate the diffusion of these ions, preventing rapid depletion and enabling prolonged antibacterial action. This synergy between controlled ionic release and polymer-mediated diffusion control represents an attractive strategy for designing multifunctional antibacterial biomaterials [41].

#### 3.4.4. Relevance for Hydrogel–Hydroxyapatite Composites

In the context of antibacterial hydrogel systems, ionic release mechanisms complement other functional contributions of hydroxyapatite. While adsorption-based drug delivery remains the dominant antibacterial pathway, ionic effects can contribute to long-term microenvironment modulation. The presence of HA particles within hydrogels may therefore support a combined strategy involving: (a) controlled antibiotic release, (b) ionic modulation of the microenvironment, and (c) improved osteoconductivity and tissue integration.

Such synergistic interactions are particularly relevant in the treatment of infected bone defects, where persistent bacterial colonization and impaired tissue regeneration often occur simultaneously. By combining drug delivery capabilities with ion-mediated biological effects, hydrogel–hydroxyapatite composites may provide more comprehensive therapeutic performance than polymeric systems alone.

### 3.5. Hydrogel–Hydroxyapatite Composites for Infected Bone Applications

The treatment of bone infections such as osteomyelitis and implant-associated infections remains a major clinical challenge. These conditions are characterized by complex interactions between bacterial biofilms, compromised vascularization, and inflammatory processes that hinder both immune clearance and antibiotic penetration. Systemic antibiotic therapy often fails to achieve sufficiently high concentrations at the infection site without inducing systemic toxicity, while surgical debridement alone rarely guarantees complete eradication of bacterial reservoirs. Consequently, localized drug delivery systems capable of providing sustained antibacterial activity while simultaneously supporting tissue regeneration have received increasing attention in recent years [51,52].

Within this context, hydrogel–hydroxyapatite composites represent a particularly promising class of biomaterials. Peptide-based hydrogel systems have also been investigated as biomimetic matrices capable of promoting hydroxyapatite nucleation and mineral growth within polymer networks [53]. Hydrogels offer a hydrated polymer network capable of encapsulating therapeutic agents and enabling controlled diffusion, whereas hydroxyapatite provides osteoconductive cues and a mineral phase structurally analogous to the inorganic component of bone. When combined, these materials create multifunctional constructs that simultaneously address infection control and bone repair—two processes that are often inseparable in clinical scenarios involving bone defects or implant-related infections [9,10,11,24,51].

#### 3.5.1. Local Antibiotic Delivery in Bone Infection

Localized antibiotic delivery has long been recognized as a powerful strategy for treating osteomyelitis. Traditional systems such as polymethylmethacrylate (PMMA) antibiotic beads have been widely used in orthopedic surgery; however, they present several limitations, including the need for secondary surgical removal, limited drug release duration, and poor integration with surrounding tissue [52]. In contrast, hydrogel-based systems provide biodegradable and injectable alternatives that can adapt to irregular defect geometries and enable more controlled release profiles.

Incorporation of hydroxyapatite into hydrogel matrices enhances these systems in several important ways. First, HA particles provide additional adsorption sites that enable higher drug loading capacities compared with purely polymeric hydrogels. Second, the adsorption–desorption interactions between antibiotics and HA surfaces can significantly prolong drug release, reducing the burst release commonly observed in hydrogel-only systems. Third, the mineral phase can improve mechanical stability and structural integrity of the composite scaffold within bone defects [11,24,42,43,44].

Several studies have demonstrated the effectiveness of HA-containing hydrogel systems for localized antibiotic delivery in bone-related infections. For example, composite scaffolds combining gelatin or chitosan hydrogels with nano-hydroxyapatite particles have shown sustained release of antibiotics such as gentamicin, vancomycin, or ciprofloxacin while maintaining antibacterial activity against common pathogens including *Staphylococcus aureus* and *Staphylococcus epidermidis* [38,54,55]. These systems not only provide antibacterial protection but also support osteoblast adhesion and mineral deposition, thereby promoting tissue regeneration alongside infection control.

#### 3.5.2. Osteoconductivity and Regenerative Support

Beyond drug delivery, the osteoconductive nature of hydroxyapatite is a central factor in the design of hydrogel–HA composites for infected bone repair. Osteoconduction refers to the ability of a material to support the attachment, migration, and proliferation of osteogenic cells along its surface. Because HA closely resembles the mineral component of natural bone, it provides favorable biochemical and structural cues for bone tissue regeneration [9,10,42].

In hydrogel composites, HA particles can serve as nucleation sites for calcium phosphate deposition and facilitate mineralization processes that lead to new bone formation. This effect is particularly important in infected bone defects, where tissue destruction and inflammation often impair natural regenerative capacity. By providing a mineral scaffold that promotes osteoblast activity and extracellular matrix deposition, HA-containing hydrogels can accelerate the healing process while simultaneously delivering antibacterial agents [24,56].

Moreover, the gradual dissolution of HA nanoparticles may release calcium and phosphate ions that further stimulate osteogenic differentiation and mineralization pathways. This ion-mediated signaling has been associated with enhanced expression of osteogenic markers such as alkaline phosphatase, osteocalcin, and collagen type I in mesenchymal stem cells and osteoblast-like cells [3,45,56].

#### 3.5.3. Composite Scaffold Architecture and Mechanical Stability

Another advantage of HA–hydrogel composites lies in their ability to combine the mechanical reinforcement provided by inorganic particles with the flexibility and permeability of polymer networks. Hydrogels alone often exhibit limited mechanical strength, which can restrict their use in load-bearing or mechanically demanding environments such as bone defects. Incorporation of HA nanoparticles can increase compressive modulus and improve structural stability without compromising the hydrated environment required for cell viability and drug diffusion [11,24,26].

The architecture of the composite scaffold plays a crucial role in determining both mechanical and biological performance. Uniform dispersion of HA nanoparticles within the hydrogel matrix is essential to prevent particle aggregation and ensure homogeneous mechanical reinforcement. Additionally, porosity and interconnected channels within the hydrogel network facilitate nutrient transport, vascularization, and infiltration of osteogenic cells. Advances in fabrication techniques—including injectable hydrogels, 3D printing, and hybrid hydrogel–electrospun constructs—have enabled the development of highly controlled architectures tailored to specific clinical needs [57,58].

#### 3.5.4. Toward Multifunctional Platforms for Bone Infection Management

The growing interest in HA–hydrogel composites reflects a broader trend toward multifunctional biomaterials capable of addressing multiple therapeutic objectives simultaneously. In the context of infected bone defects, an ideal material should integrate several functions: (a) localized antibacterial delivery, (b) biofilm suppression, (c) structural support, (d) promotion of bone regeneration, and (e) compatibility with minimally invasive implantation.

Hydrogel–hydroxyapatite composites offer a platform capable of fulfilling many of these requirements. Furthermore, they can be readily integrated with additional functional components—including bacteriophages, antimicrobial peptides, or electrospun reinforcing networks—thereby aligning with the tri-pillar framework proposed in this review. Such hybrid systems may enable synergistic strategies where biological antibacterial agents, inorganic drug reservoirs, and structural scaffolds work together to enhance therapeutic outcomes [8,27,29].

**Figure 6:**
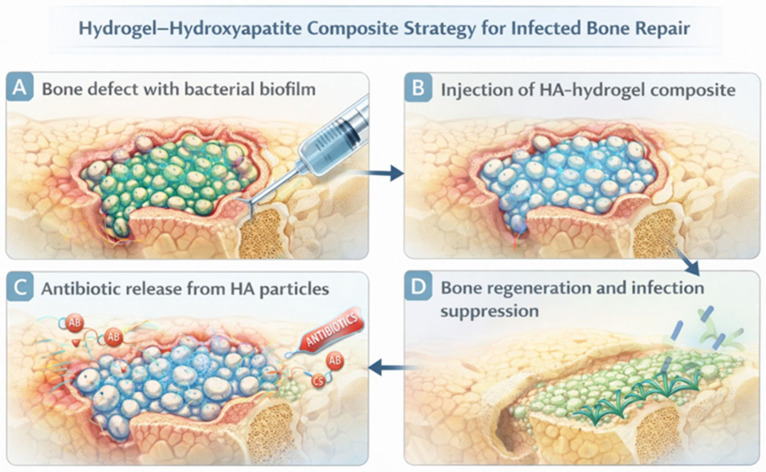
Hydrogel–hydroxyapatite composite strategy for the treatment of infected bone defects. (**A**) Schematic representation of a bone defect colonized by bacterial biofilm. (**B**) Injection or implantation of a hydrogel–hydroxyapatite composite into the defect site. (**C**) Localized release of antibiotics from hydroxyapatite particles dispersed within the hydrogel matrix, enabling sustained antibacterial activity. (**D**) Combined infection suppression and bone regeneration supported by the osteoconductive properties of hydroxyapatite and the structural environment provided by the hydrogel network.

The conceptual strategy for using hydrogel–hydroxyapatite composites in the treatment of infected bone defects is schematically illustrated in Figure 6.

Despite these promising developments, challenges remain. Optimization of HA particle size, surface properties, and concentration is necessary to balance drug loading capacity with mechanical performance and injectability. In addition, more in vivo and clini cal studies are required to establish standardized protocols and evaluate long-term outcomes in bone infection treatment [52,57].

Overall, hydrogel–hydroxyapatite composites represent a powerful and versatile approach for the treatment of bone infections, combining controlled antibacterial delivery with regenerative functionality in a single integrated biomaterial platform.

### 3.6. Integration Within Multifunctional Hydrogel Platforms

The development of antibacterial hydrogel systems increasingly relies on multifunctional design strategies capable of integrating complementary mechanisms within a single material platform. In this context, hydroxyapatite-containing hydrogels represent a particularly versatile framework because the inorganic phase can simultaneously contribute to structural reinforcement, drug adsorption, ionic modulation of the local microenvironment, and regenerative support. However, the full potential of these systems becomes most apparent when hydroxyapatite is incorporated into hybrid platforms that combine biological antibacterial agents and advanced structural architectures.

**Figure 7:**
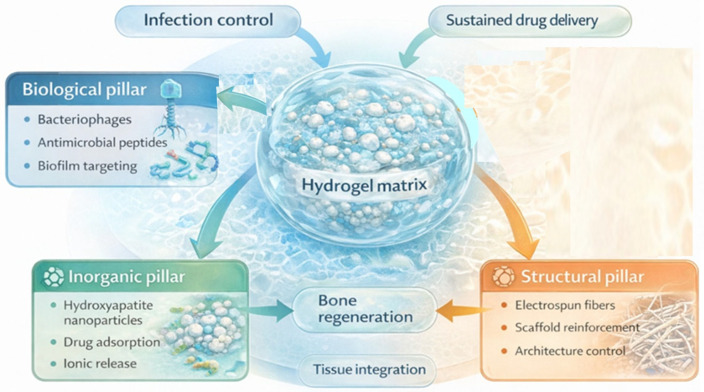
Conceptual integration of the three pillars in multifunctional antibacterial hydrogel platforms. Schematic representation of hybrid hydrogel systems combining biological antibacterial agents (e.g., bacteriophages or antimicrobial peptides), inorganic components such as hydroxyapatite nanoparticles acting as drug reservoirs and ionic modulators, and structural architectures including electrospun fibrous scaffolds. The integration of these three pillars enables simultaneous infection control, sustained drug delivery, mechanical reinforcement, and tissue regeneration within advanced multifunctional biomaterial platforms.

Within the tri-pillar framework proposed in this review, hydroxyapatite serves as the inorganic component that bridges biological functionality and structural engineering. While bacteriophages and other biological agents provide highly specific antibacterial activity, and electrospun or architectured polymer networks supply structural guidance, hydroxyapatite contributes physicochemical functionality that stabilizes therapeutic payloads and promotes tissue integration. The convergence of these three elements enables the development of next-generation biomaterials designed not only to eliminate infection but also to support tissue regeneration and long-term implant integration. The conceptual integration of biological, inorganic, and structural strategies within multifunctional hydrogel systems is illustrated in Figure 7.

#### 3.6.1. Integration with Biological Antibacterial Agents

One promising direction involves combining hydroxyapatite-based hydrogel systems with biologically derived antibacterial agents such as bacteriophages, antimicrobial peptides, or phage-derived enzymes. In these systems, hydroxyapatite may act as a stabilizing carrier or adsorption platform that enhances the retention of biological agents within the hydrogel matrix. For instance, electrostatic interactions between viral capsid proteins and mineral surfaces may influence the spatial distribution and persistence of bacteriophages in composite materials, potentially prolonging antibacterial activity at the infection site [59].

Moreover, the mineral phase may indirectly protect biological agents from rapid diffusion or degradation by providing adsorption sites and modifying local ionic conditions. Although the interaction between bacteriophages and hydroxyapatite has not been extensively explored, preliminary studies suggest that mineral surfaces can influence phage stability and adsorption dynamics, opening new possibilities for hybrid antibacterial systems that combine biological specificity with inorganic functionality [33,60].

#### 3.6.2. Integration with Advanced Structural Architectures

Beyond biological integration, hydroxyapatite-containing hydrogels can also be combined with advanced structural scaffolds that provide mechanical reinforcement and spatial organization. Electrospun nanofiber networks, for example, can be embedded within hydrogel matrices to create composite systems that mimic the hierarchical architecture of natural extracellular matrices. Such structures offer improved mechanical stability, increased surface area for cell attachment, and enhanced control over drug release pathways [61].

In these hybrid systems, hydroxyapatite nanoparticles may interact with both the hydrogel phase and the fibrous scaffold, creating interconnected networks that improve load transfer and structural cohesion. The presence of mineral particles can also influence fiber–hydrogel interfacial interactions, potentially enhancing mechanical performance while preserving the diffusion properties required for controlled drug delivery.

Recent developments in additive manufacturing and biofabrication techniques further expand the possibilities for integrating hydroxyapatite within multifunctional hydrogel platforms. Three-dimensional printing approaches enable precise spatial distribution of mineral particles and therapeutic agents within complex scaffold geometries, allowing researchers to tailor antibacterial activity and regenerative performance to specific clinical scenarios [62].

#### 3.6.3. Toward Next-Generation Antibacterial Biomaterials

The integration of hydroxyapatite within multifunctional hydrogel platforms reflects a broader trend in biomaterials research toward systems capable of performing multiple therapeutic functions simultaneously. Rather than relying on a single antibacterial mechanism, emerging designs combine complementary strategies including: (a) localized drug delivery, (b) ionic microenvironment modulation, (c) biological antibacterial agents, and (d) structural reinforcement and regenerative support.

Such integrated systems may prove particularly valuable in the treatment of complex infections such as implant-associated infections or chronic osteomyelitis, where bacterial persistence, biofilm formation, and tissue damage occur simultaneously.

Future research should focus on understanding the interactions between these different functional components and optimizing the balance between antibacterial efficacy, mechanical stability, and biological compatibility. In particular, deeper insight into the physicochemical interactions between hydroxyapatite surfaces, biological agents, and polymer networks may enable the rational design of hybrid materials capable of achieving synergistic therapeutic effects.

Ultimately, hydroxyapatite-containing hydrogels should be viewed not simply as drug carriers but as adaptable platforms for the integration of multiple antibacterial and regenerative strategies. Within the tri-pillar framework proposed in this review, these systems represent a key step toward the development of next-generation biomaterials capable of addressing the complex challenges posed by antibiotic-resistant infections and tissue regeneration.

## 4. Structural Pillar: Electrospun Architectures in Antibacterial Hydrogel Platforms

While biological agents provide antibacterial specificity and inorganic phases contribute physicochemical functionality, the structural organization of biomaterials plays an equally critical role in determining therapeutic performance. In hydrogel-based antibacterial systems, architecture influences mass transport, mechanical behavior, cellular interactions, and the spatial distribution of therapeutic agents. Consequently, structural design has emerged as a key parameter in the development of next-generation multifunctional biomaterials.

Among the different fabrication strategies available for controlling material architecture, electrospinning has gained particular prominence. Electrospinning enables the production of continuous polymeric fibers with diameters ranging from several micrometers down to the nanoscale, generating fibrous networks that closely resemble the structural organization of native extracellular matrices [63]. These fibrous assemblies exhibit high surface-to-volume ratios, tunable porosity, and interconnected structures that facilitate cell infiltration and nutrient diffusion.

When combined with hydrogel matrices, electrospun fibers can significantly enhance the mechanical stability and functional versatility of composite biomaterials. Electrospun scaffolds may serve as reinforcing frameworks within hydrated polymer networks, improving structural integrity while preserving the diffusion properties required for controlled release of antibacterial agents. Similar hybrid wound dressing systems combining electrospun fibers with antibiotic-loaded hydrogels have been reported to provide both antimicrobial activity and favorable conditions for tissue regeneration [64].

The integration of electrospun architectures within antibacterial hydrogel systems is particularly relevant for applications involving wound healing and bone regeneration, where both structural support and biological compatibility are essential. Electrospun fibers can be engineered to incorporate antibacterial molecules directly within their structure or to serve as reservoirs for therapeutic agents within surrounding hydrogel matrices. In addition, their nanoscale morphology provides a large interfacial area that can influence bacterial adhesion, cell attachment, and tissue integration [65].

Within the tri-pillar framework proposed in this review, electrospun materials represent the structural pillar, complementing the biological and inorganic strategies described in the previous sections. While bacteriophages or other antimicrobial agents provide targeted antibacterial activity and hydroxyapatite nanoparticles contribute physicochemical functionality and regenerative potential, electrospun architectures supply the structural organization necessary to stabilize these components within multifunctional biomaterial systems.

The following subsections examine the principles of electrospinning, the design of antibacterial electrospun scaffolds, and the development of hybrid electrospun–hydrogel systems capable of integrating structural, biological, and inorganic functionalities.

### 4.1. Fundamentals of Electrospinning for Biomedical Materials

Electrospinning is a versatile fiber fabrication technique that uses electrostatic forces to produce ultrafine polymer fibers from viscous solutions or melts. The process involves applying a high-voltage electric field between a polymer solution contained in a syringe and a grounded collector. When the electrostatic forces overcome the surface tension of the polymer droplet at the needle tip, a charged jet is ejected and undergoes elongation and solvent evaporation, resulting in the formation of continuous fibers that deposit on the collector as a nonwoven mat [63].

The morphology and properties of electrospun fibers are influenced by numerous parameters, including polymer concentration, solvent properties, applied voltage, flow rate, and collector geometry. Careful control of these variables enables the fabrication of fibers with tailored diameters, alignment, and porosity. In biomedical applications, these characteristics are particularly important because they influence cell attachment, tissue infiltration, and diffusion of therapeutic molecules.

One of the most attractive features of electrospinning is the ability to produce fibrous structures that mimic the architecture of natural extracellular matrices. Native tissues such as skin, bone, and cartilage contain fibrous protein networks—primarily collagen and elastin—that provide structural support and regulate cell behavior. Electrospun polymer scaffolds can replicate these fibrous morphologies at comparable length scales, thereby creating favorable environments for tissue regeneration [64].

Electrospinning also offers multiple strategies for incorporating therapeutic agents within fibers. Antibacterial compounds can be blended directly into the polymer solution prior to spinning, resulting in homogeneous distribution throughout the fibers. Alternatively, more sophisticated approaches such as coaxial electrospinning can generate core–shell structures that enable controlled release of bioactive molecules. These strategies allow electrospun materials to function simultaneously as structural scaffolds and therapeutic delivery systems.

**Figure 8:**
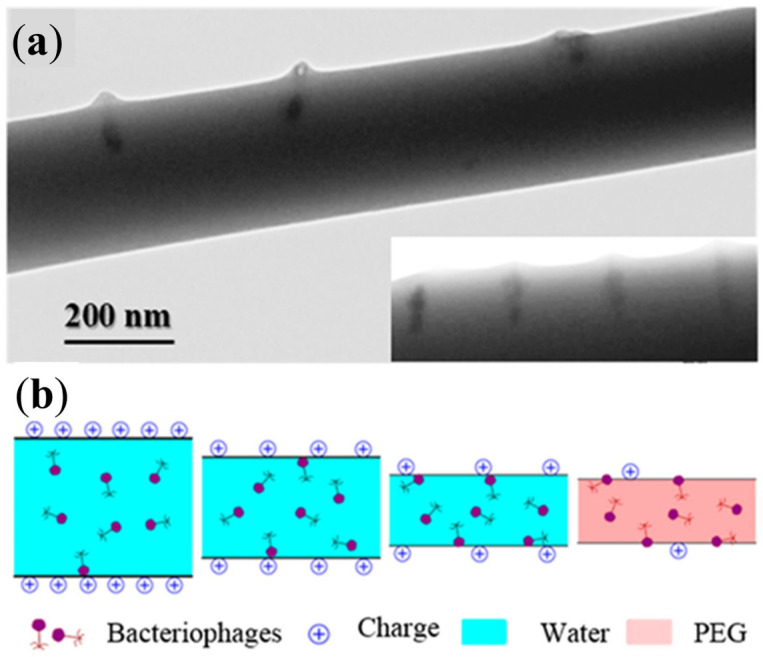
Interaction between bacteriophages and electrospun polymer microfibers. (**a**) Transmission electron microscopy (TEM) images showing the preferential adsorption and orientation of bacteriophages along the inner surface of electrospun poly(ethylene glycol) (PEG) microfibers obtained from aqueous polymer solutions. The viral particles appear aligned along the fiber walls, suggesting strong interfacial interactions between the bacteriophages and the polymer matrix. (**b**) Schematic representation of the proposed mechanism explaining the preferential distribution of bacteriophages on the fiber surface, highlighting the role of surface charge distribution on PEG microfibers in promoting phage adhesion and orientation. Reproduced with permission from [68] Díaz et al., Fibers (MDPI), distributed under the Creative Commons CC-BY license.

The large surface area of electrospun fibers further enhances their potential for antibacterial applications. Increased surface exposure can facilitate adsorption of therapeutic molecules, interaction with bacterial cells, and modulation of biofilm formation. When electrospun mats are combined with hydrogel systems, the resulting composites may exhibit improved mechanical stability and more controlled release profiles compared with either material alone [61,65,66,67]. Experimental studies have demonstrated that bacteriophages can be successfully incorporated into electrospun polymer fibers while preserving their antibacterial activity [68]. Experimental observations have also shown that bacteriophages can preferentially adsorb and orient along electrospun polymer microfibers, a phenomenon influenced by surface charge distribution and interfacial interactions between the viral particles and the polymer matrix (Figure 8).

**Figure 9:**
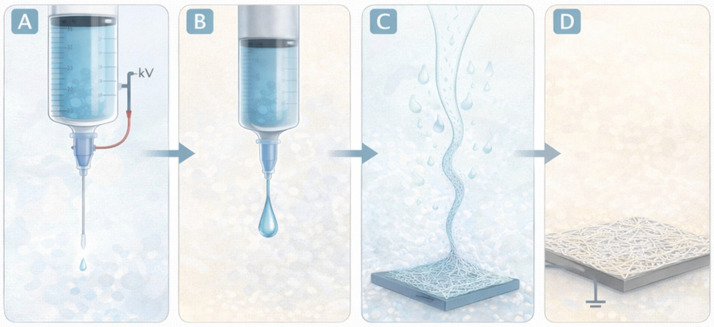
Schematic representation of the electrospinning process and formation of nanofibrous scaffolds. (**A**) Polymer solution contained in a syringe connected to a high-voltage power supply. (**B**) Formation of the Taylor cone at the needle tip as electrostatic forces overcome surface tension. (**C**) Elongation and whipping of the charged polymer jet accompanied by solvent evaporation, leading to the formation of ultrafine fibers. (**D**) Deposition of continuous nanofibers onto a grounded collector, producing a nonwoven fibrous scaffold.

For these reasons, electrospinning has become one of the most widely used techniques for designing fibrous biomaterials in regenerative medicine, wound healing, and drug delivery applications. The fundamental stages of the electrospinning process and the formation of nanofibrous scaffolds are schematically illustrated in Figure 9.

### 4.2. Antibacterial Electrospun Fibrous Systems

Electrospun nanofibrous materials have emerged as highly promising platforms for antibacterial applications due to their unique structural and physicochemical characteristics [69]. The high surface-to-volume ratio, interconnected porosity, and tunable fiber morphology of electrospun scaffolds create favorable conditions for both the incorporation and controlled release of antibacterial agents. In addition, the nanoscale architecture of electrospun fibers closely resembles the extracellular matrix of natural tissues, which supports cell adhesion, migration, and tissue regeneration. These combined properties make electrospun systems particularly attractive for biomedical applications requiring both antimicrobial activity and structural support, such as wound healing, implant coatings, and bone tissue engineering [70,71,72]. More broadly, electrospinning has been extensively explored as a versatile platform for the fabrication of functional biomaterials across a wide range of biomedical applications, highlighting its adaptability in terms of material selection, structural design, and therapeutic integration [73].

**Figure 10:**
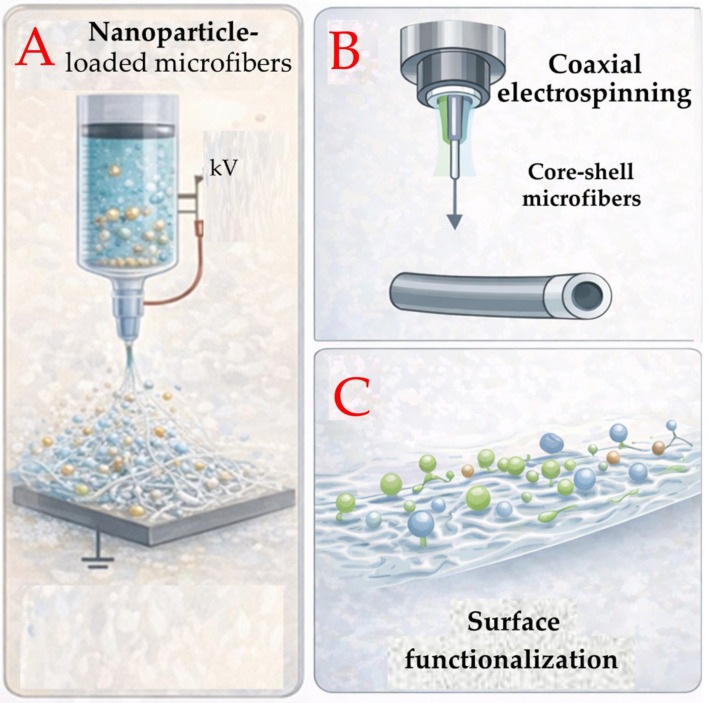
Strategies for incorporating antibacterial agents into electrospun fibers. Schematic representation of the principal approaches used to introduce antibacterial functionality into electrospun scaffolds. (**A**) Nanoparticle-loaded fibers, where antimicrobial nanoparticles are incorporated within the polymer matrix during electrospinning. (**B**) Coaxial electrospinning, producing core–shell fibers in which antibacterial compounds are encapsulated in the core and protected by an outer polymer shell, enabling controlled release. (**C**) Surface functionalization, where antimicrobial molecules or nanoparticles are immobilized on the fiber surface through post-processing techniques.

A key advantage of electrospinning is the versatility with which antibacterial agents can be incorporated into fibrous scaffolds. Antimicrobial compounds may be blended directly with polymer solutions prior to spinning, embedded within core–shell fibers using coaxial electrospinning, or immobilized on the fiber surface through post-processing functionalization. Each of these strategies allows for different control over drug loading, release kinetics, and antibacterial performance, enabling the design of scaffolds tailored to specific therapeutic requirements [74]. Melt electrospinning approaches have also been investigated for the incorporation of antibacterial compounds into polymer scaffolds while maintaining sustained release behavior [75]. The main strategies for incorporating antibacterial agents into electrospun fibers are schematically summarized in Figure 10. Beyond structural design and drug incorporation strategies, recent research has increasingly focused on the development of electrospun systems with enhanced biological functionality and improved integration within complex tissue environments. In particular, efforts have been directed toward the incorporation of bioactive molecules, peptides, and multifunctional agents capable of simultaneously promoting antibacterial activity and tissue regeneration. These approaches highlight the potential of electrospun scaffolds as dynamic therapeutic platforms rather than passive delivery systems. However, such increased functional complexity also introduces additional challenges related to the stability of sensitive biomolecules during processing, as well as the need for precise control over fiber architecture and degradation behavior to ensure predictable in vivo performance. Recent advances in electrospinning techniques have further emphasized that, despite their versatility, the successful translation of electrospun biomedical systems depends on improved process control, reproducibility, and compatibility with sensitive therapeutic cargos, underscoring the importance of robust fabrication strategies for clinically relevant antibacterial platforms [76]. In addition, large-scale production remains a major bottleneck for electrospinning-based systems, since maintaining fiber uniformity, process stability, and acceptable production yields under industrial conditions is considerably more complex than under laboratory-scale settings [77].

Electrospun antibacterial systems have been developed using a wide range of polymers, including both natural and synthetic materials. Natural polymers such as chitosan, gelatin, collagen, and silk fibroin provide inherent biocompatibility and may exhibit intrinsic antibacterial or bioactive properties. Synthetic polymers such as poly(ε-caprolactone) (PCL), poly(lactic acid) (PLA), and poly(lactic-co-glycolic acid) (PLGA) offer improved mechanical stability and controllable degradation rates. Blends of natural and synthetic polymers are frequently employed to combine biological functionality with structural robustness [71,78]. Electrospun poly(lactic acid) scaffolds loaded with antibiotics have also demonstrated controlled release behavior and significant antibacterial activity against pathogenic bacteria [79].

#### 4.2.1. Strategies for Incorporating Antibacterial Agents

Several approaches have been developed for incorporating antibacterial functionality into electrospun fibers. The simplest strategy involves blending antimicrobial agents directly into the polymer solution before electrospinning. In this approach, antibacterial molecules become distributed throughout the fiber structure and are gradually released as the polymer matrix degrades or through diffusion processes. This method has been widely applied for the incorporation of antibiotics such as tetracycline, ciprofloxacin, and gentamicin within electrospun scaffolds [80].

More advanced architectures can be achieved through coaxial electrospinning, which produces fibers with a core–shell structure [81]. In such systems, antibacterial agents may be confined within the core region while the outer shell controls diffusion and protects sensitive molecules from environmental degradation. Core–shell electrospun fibers have demonstrated improved control over release profiles and reduced initial burst release compared with conventional blended systems [74,82].

Surface functionalization represents another important strategy for imparting antibacterial properties. In this approach, electrospun fibers are modified after fabrication by immobilizing antimicrobial molecules or nanoparticles onto the fiber surface. Techniques such as plasma treatment, chemical grafting, and layer-by-layer deposition have been employed to introduce antibacterial functionalities without compromising fiber morphology [83].

#### 4.2.2. Electrospun Fibers Incorporating Antibacterial Nanomaterials

In addition to conventional antibiotics, a variety of inorganic nanomaterials have been incorporated into electrospun fibers to achieve antibacterial activity. Silver nanoparticles, zinc oxide nanoparticles, and copper-based nanomaterials are among the most widely investigated systems due to their broad-spectrum antimicrobial properties. These nanoparticles can disrupt bacterial membranes, generate reactive oxygen species, and interfere with essential metabolic pathways within microbial cells [84,85].

Electrospun nanofibers provide an ideal platform for stabilizing and distributing such nanoparticles. The fibrous matrix prevents nanoparticle aggregation while enabling controlled release of antimicrobial ions. Furthermore, the large surface area of electrospun fibers enhances contact between antibacterial agents and bacterial cells, improving overall antimicrobial efficiency.

Hybrid systems combining nanoparticles with antibiotics or bioactive molecules have also been explored to achieve synergistic antibacterial effects. For example, electrospun scaffolds incorporating both silver nanoparticles and antibiotics have demonstrated enhanced activity against multidrug-resistant bacterial strains [85].

#### 4.2.3. Applications in Wound Healing and Tissue Regeneration

Electrospun antibacterial scaffolds have been extensively investigated for wound healing applications. The fibrous morphology of electrospun materials mimics the structural organization of the extracellular matrix in skin tissue, supporting keratinocyte and fibroblast proliferation while maintaining adequate moisture levels at the wound site. When combined with antibacterial agents, these scaffolds can simultaneously prevent infection and promote tissue repair [86].

In bone tissue engineering, electrospun fibers have been incorporated into composite scaffolds containing hydroxyapatite nanoparticles or bioactive ceramics to enhance osteoconductivity and mechanical stability. Such systems provide structural reinforcement while enabling localized delivery of antibacterial agents to prevent infection during bone regeneration processes [87].

The integration of electrospun fibrous scaffolds with hydrogel matrices further expands the potential of these systems. Electrospun networks can act as reinforcing frameworks within hydrogels, improving mechanical stability and providing hierarchical architectures that combine the advantages of fibrous scaffolds and hydrated polymer networks. These hybrid materials are particularly promising for applications requiring both structural integrity and sustained antibacterial activity [65,87].

#### 4.2.4. Challenges and Future Directions

Despite the versatility of electrospinning, several intrinsic limitations must be considered. The requirement for high voltage and organic solvents may compromise the stability of sensitive biological agents such as bacteriophages. In addition, achieving homogeneous incorporation of therapeutic agents within nanofibers remains challenging, particularly for hydrophilic compounds. Scale-up of electrospinning processes also presents significant technical barriers, as reproducibility and production rates are often limited compared with conventional manufacturing methods.

In addition, several challenges specifically remain in the development of electrospun antibacterial scaffolds. Achieving precise control over drug loading and release kinetics remains a major challenge, particularly for highly soluble antibacterial agents that may exhibit rapid initial release. In addition, the long-term stability and potential cytotoxicity of certain antibacterial nanoparticles must be carefully evaluated before clinical translation.

Despite the significant advantages of electrospinning for the fabrication of antibacterial fibrous systems, several critical limitations must be carefully considered for their effective implementation in hydrogel-based platforms. In particular, the incorporation of biologically active agents such as bacteriophages presents challenges related to process-induced stress, including exposure to high voltage, shear forces, and solvent environments that may compromise biological activity. Furthermore, while electrospun architectures offer high surface area and tunable porosity, they may also lead to heterogeneous distribution of active agents and uncontrolled initial burst release. From a manufacturing perspective, scalability and reproducibility remain key challenges, as the transition from laboratory-scale electrospinning to industrial production is still limited by low throughput and process variability. Additionally, the integration of electrospun fibers within hydrogel matrices introduces further complexity in terms of interfacial stability, mechanical compatibility, and long-term structural integrity. Addressing these limitations will be essential for the successful translation of electrospun hydrogel-based antibacterial systems into clinical applications. In this context, the development of hybrid fabrication approaches and mild processing strategies is expected to play a crucial role in overcoming current limitations.

Future research is expected to focus on the development of multifunctional electrospun systems capable of integrating antibacterial activity with regenerative functionality. Strategies such as stimuli-responsive release, bioactive surface modification, and the incorporation of biological agents—including bacteriophages—may further enhance the therapeutic potential of electrospun scaffolds.

Within the context of the tri-pillar framework proposed in this review, electrospun antibacterial systems represent a critical structural component capable of stabilizing and organizing biological and inorganic antibacterial strategies within multifunctional hydrogel platforms.

### 4.3. Electrospun–Hydrogel Hybrid Systems

Hybrid materials combining electrospun fibrous scaffolds with hydrogel matrices have emerged as highly promising platforms for biomedical applications requiring both structural organization and controlled drug delivery [88]. These composite systems integrate the mechanical stability and extracellular matrix–like architecture of electrospun fibers with the high water content and diffusion-controlled release properties of hydrogels. As a result, electrospun–hydrogel hybrids can overcome many of the limitations associated with either material alone, providing multifunctional environments suitable for antibacterial therapy and tissue regeneration [89,90].

**Figure 11:**
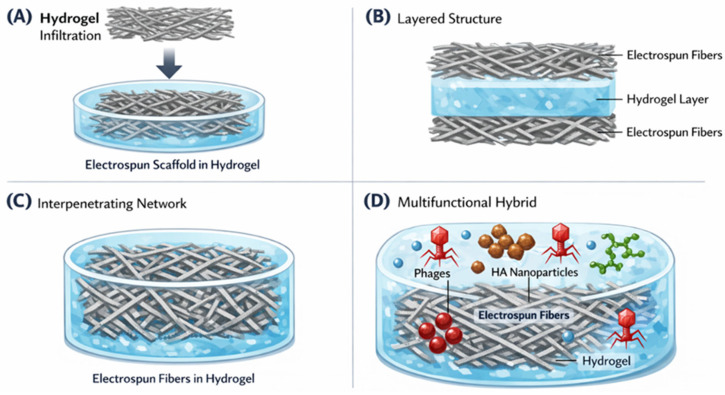
Schematic representation of different strategies used to integrate electrospun fibrous scaffolds with hydrogel matrices. (**A**) Hydrogel infiltration into a pre-formed electrospun scaffold. (**B**) Layered electrospun–hydrogel composite structures. (**C**) Electrospun fibers embedded within a hydrogel matrix forming interpenetrating networks. (**D**) Multifunctional hybrid systems integrating electrospun fibers, hydrogel matrices, and bioactive components such as bacteriophages, hydroxyapatite nanoparticles, and antibacterial molecules.

Hydrogels are widely used as drug delivery platforms due to their high swelling capacity and ability to encapsulate therapeutic agents within hydrated polymer networks. However, their mechanical strength is often limited, particularly in applications involving load-bearing tissues or mechanically demanding environments. Electrospun fibrous scaffolds can address this limitation by acting as reinforcing frameworks within the hydrogel matrix. The resulting composite materials combine the elasticity and permeability of hydrogels with the structural stability of fibrous networks, generating hybrid architectures with improved mechanical performance and tunable drug release behavior [91]. Different structural strategies for combining electrospun fibrous scaffolds with hydrogel matrices are schematically illustrated in Figure 11.

#### 4.3.1. Structural Integration of Fibrous Scaffolds and Hydrogels

Several strategies have been developed for integrating electrospun fibers with hydrogel matrices. One common approach involves infiltrating a pre-formed electrospun scaffold with a hydrogel precursor solution, followed by in situ crosslinking of the polymer network. In this configuration, the electrospun scaffold acts as a three-dimensional framework that reinforces the hydrogel while preserving its porous structure. This approach enables the fabrication of composites that mimic the hierarchical organization of natural tissues, where fibrous proteins such as collagen are embedded within hydrated extracellular matrices [90,92].

Alternatively, electrospun fibers can be deposited directly onto hydrogel surfaces or layered between hydrogel sheets to produce multilayer structures with controlled mechanical and biological properties. Such layered architectures may improve structural integrity while enabling spatial separation of different therapeutic agents within the composite system. For example, antibacterial compounds may be incorporated within electrospun layers while growth factors or regenerative molecules are embedded within the hydrogel phase [93].

More advanced fabrication strategies involve simultaneous electrospinning and hydrogel formation processes. In these systems, electrospun fibers are generated while hydrogel networks form around them, resulting in interpenetrating structures where both phases are intimately integrated. These approaches can produce highly uniform hybrid materials with improved interfacial bonding between fibers and polymer networks [91].

#### 4.3.2. Drug Delivery and Antibacterial Functionality

Electrospun–hydrogel hybrid systems provide multiple opportunities for incorporating antibacterial agents and controlling their release. Therapeutic molecules may be loaded into electrospun fibers, encapsulated within hydrogel matrices, or distributed across both components to create hierarchical delivery systems. Such dual-reservoir architectures allow for the design of complex release profiles that combine rapid initial antibacterial action with prolonged therapeutic activity.

For instance, antibiotics incorporated within electrospun fibers may provide an initial burst release that rapidly reduces bacterial load at the infection site, while hydrogels containing additional antibacterial agents sustain therapeutic concentrations over longer periods. This complementary release behavior can be particularly beneficial for preventing bacterial colonization during the early stages of wound healing or implant integration [94].

Hybrid electrospun–hydrogel systems also facilitate the incorporation of diverse antibacterial agents, including antibiotics, antimicrobial peptides, metallic nanoparticles, and biological agents such as bacteriophages. The fibrous component may protect sensitive molecules from rapid degradation, while the hydrogel matrix provides a hydrated environment that preserves biological activity. Such multifunctional delivery platforms are increasingly being investigated for the treatment of antibiotic-resistant infections [95].

#### 4.3.3. Mechanical Reinforcement and Structural Stability

The incorporation of electrospun fibers significantly improves the mechanical properties of hydrogel-based systems. Hydrogels alone often exhibit limited tensile strength and structural stability, which can restrict their use in certain biomedical applications. Electrospun fiber networks can reinforce the hydrogel matrix by distributing mechanical stress throughout the structure, thereby increasing stiffness, tensile strength, and resistance to deformation [91].

The degree of mechanical reinforcement depends on several factors, including fiber orientation, fiber density, and interfacial bonding between fibers and hydrogel polymers. Aligned electrospun fibers, for example, may enhance anisotropic mechanical properties and guide cellular growth along preferred directions. Random fibrous networks, on the other hand, provide isotropic reinforcement that improves overall structural integrity.

Such reinforcement is particularly important for applications involving tissue regeneration in mechanically demanding environments, such as bone repair or cartilage regeneration. Hybrid electrospun–hydrogel systems can maintain structural integrity while supporting cell infiltration and tissue remodeling, making them attractive candidates for regenerative medicine applications [96].

#### 4.3.4. Applications in Wound Healing and Tissue Engineering

Electrospun–hydrogel composites have been widely investigated in wound healing applications due to their ability to combine antibacterial protection with favorable environments for tissue regeneration. The fibrous component mimics the extracellular matrix of skin tissue, promoting cell adhesion and migration, while the hydrogel phase maintains moisture and enables sustained delivery of antibacterial agents. Such materials may reduce infection risk while accelerating wound closure and regeneration [86,97].

In bone tissue engineering, electrospun–hydrogel hybrids can be combined with hydroxyapatite nanoparticles to produce composite scaffolds that support both osteogenesis and antibacterial activity. These systems integrate the structural reinforcement provided by electrospun fibers, the osteoconductive properties of hydroxyapatite, and the drug delivery capabilities of hydrogels. As discussed in previous sections, such multifunctional platforms may be particularly effective in treating infected bone defects where both bacterial eradication and tissue regeneration are required [11,24,96].

#### 4.3.5. Emerging Multifunctional Architectures

Recent advances in fabrication technologies are enabling increasingly sophisticated electrospun–hydrogel hybrid systems. Three-dimensional printing, microfluidic electrospinning, and dynamic crosslinking strategies are being explored to create hierarchical biomaterials with spatially controlled architectures and programmable release profiles. These approaches allow for precise control over the distribution of fibers, hydrogels, and therapeutic agents within complex scaffold geometries.

Such developments are driving the evolution of electrospun–hydrogel composites toward multifunctional platforms capable of integrating antibacterial therapy with regenerative medicine strategies. Within the tri-pillar framework proposed in this review, these hybrid systems represent the structural backbone that supports the incorporation of biological antibacterial agents and inorganic functional components, enabling the development of next-generation biomaterials designed to address complex infectious diseases.

### 4.4. Integration with Biological and Inorganic Strategies

Beyond their structural and delivery capabilities, electrospun–hydrogel hybrid systems can serve as platforms for integrating biological and inorganic antibacterial strategies. Their full potential becomes evident when these structural frameworks are combined with complementary functional components. As discussed in previous sections, bacteriophages provide highly specific antibacterial activity, while hydroxyapatite-based materials contribute physicochemical functionality and osteoconductive properties. Electrospun architectures, in turn, supply the structural framework necessary to stabilize these functional components within complex biomaterial systems.

Within this context, electrospun–hydrogel composites can act as multifunctional integration platforms, capable of hosting diverse antibacterial agents while maintaining controlled release profiles and appropriate mechanical properties. The fibrous component provides a large surface area for the adsorption or immobilization of bioactive molecules, whereas the hydrogel matrix offers a hydrated environment that facilitates diffusion and preserves the biological activity of sensitive therapeutic agents [89,98]. Recent studies have explored multifunctional biomaterial platforms combining polymer scaffolds with bioactive nanoparticles and antibacterial agents to simultaneously address infection control and tissue regeneration [24].

#### 4.4.1. Integration with Bacteriophage-Based Antibacterial Systems

Electrospun–hydrogel hybrid materials offer particularly attractive opportunities for the incorporation of bacteriophages. The fibrous architecture of electrospun scaffolds can provide protective microenvironments that help maintain phage stability, while the surrounding hydrogel phase enables sustained diffusion and delivery of viral particles toward bacterial targets.

Several studies have demonstrated that polymeric matrices can effectively encapsulate bacteriophages without significantly compromising their infectivity. In hybrid systems, electrospun fibers may act as structural reservoirs that retain phages within the scaffold, while hydrogel matrices regulate their release and distribution in the surrounding environment. Such dual-phase delivery systems could be especially advantageous for treating biofilm-associated infections, where localized and sustained antibacterial activity is required [33,99].

Moreover, the high porosity of electrospun scaffolds may facilitate bacterial access to embedded phages, potentially improving the efficiency of phage–bacteria interactions. These properties suggest that electrospun–hydrogel composites may represent promising platforms for the development of phage-based antibacterial biomaterials, particularly in applications involving wound healing or implant-associated infections.

#### 4.4.2. Integration with Hydroxyapatite-Based Functional Components

The incorporation of hydroxyapatite nanoparticles into electrospun–hydrogel systems further expands the multifunctional capabilities of these materials. As discussed in Section 3, hydroxyapatite particles can act as reservoirs for therapeutic agents, contribute to ionic microenvironment modulation, and promote osteoconductive behavior in bone regeneration contexts.

In hybrid electrospun–hydrogel scaffolds, hydroxyapatite nanoparticles may be incorporated within electrospun fibers, dispersed within hydrogel matrices, or distributed throughout both components. Such configurations enable the design of hierarchical biomaterials in which structural reinforcement, drug delivery, and regenerative functions are simultaneously integrated.

These composite systems are particularly relevant for orthopedic applications, where the combination of antibacterial activity and bone regeneration is essential. Electrospun fibers may provide mechanical stability and guide cell attachment, hydrogels enable controlled release of therapeutic agents, and hydroxyapatite nanoparticles support osteogenic processes. The resulting multifunctional platforms therefore represent promising candidates for the treatment of infected bone defects and implant-associated infections [29,100].

#### 4.4.3. Toward Integrated Multifunctional Biomaterials

The convergence of electrospinning, hydrogel chemistry, and bioactive inorganic components reflects a broader trend in biomaterials research toward the development of integrated multifunctional systems. Rather than relying on a single antibacterial mechanism, emerging strategies seek to combine complementary approaches that address multiple aspects of infection and tissue regeneration simultaneously.

Within the tri-pillar framework proposed in this review, electrospun–hydrogel hybrids function as structural platforms capable of organizing biological antibacterial agents and inorganic functional components within hierarchical architectures. Such systems may provide synergistic therapeutic effects by combining targeted antibacterial activity, controlled drug release, and regenerative support within a single material platform.

Future research in this field will likely focus on optimizing the interactions between these different functional elements, improving fabrication techniques for complex hybrid architectures, and translating these multifunctional materials toward clinically relevant applications.

## 5. Challenges and Future Perspectives

Despite the considerable progress achieved in the development of antibacterial hydrogel systems, several scientific and technological challenges remain before these materials can be translated into routine biomedical applications. The integration of biological agents, inorganic functional components, and advanced structural architectures offers promising opportunities for addressing antibiotic-resistant infections. However, the complexity of these multifunctional systems also introduces new challenges related to stability, scalability, and clinical implementation.

### 5.1. Stability and Activity of Biological Antibacterial Agents

One of the most critical challenges concerns the stability and long-term activity of biological antibacterial agents, particularly bacteriophages. Although phage therapy has gained renewed interest in recent years due to the increasing prevalence of antibiotic-resistant pathogens, maintaining phage viability within polymeric matrices remains a significant issue. Environmental conditions such as pH, temperature, dehydration, and interactions with polymer components may affect phage stability and infectivity during storage and application [101].

Hydrogel-based systems can provide protective microenvironments that help preserve biological activity, but additional strategies may be required to optimize phage encapsulation and controlled release. Approaches such as microencapsulation, protective polymer coatings, or stabilization through adsorption onto inorganic particles may enhance phage persistence in complex biomaterial platforms. Future research should focus on understanding the interactions between bacteriophages and polymeric matrices to ensure reliable and reproducible antibacterial performance.

### 5.2. Control of Drug Release and Therapeutic Efficiency

Another major challenge lies in achieving precise control over the release of antibacterial agents from multifunctional hydrogel systems. Although electrospun–hydrogel composites can offer sophisticated drug delivery capabilities, the simultaneous incorporation of multiple therapeutic components may complicate release kinetics and diffusion pathways. Interactions between polymer networks, inorganic particles, and therapeutic agents can significantly influence release profiles, potentially leading to undesired burst release or incomplete drug delivery [102].

Advanced strategies such as stimuli-responsive hydrogels, dynamic crosslinking networks, and multi-compartment drug delivery systems may provide improved control over therapeutic release. These approaches could enable responsive biomaterials capable of adapting drug delivery to environmental conditions such as pH changes, enzymatic activity, or bacterial presence.

### 5.3. Manufacturing and Scalability

The translation of multifunctional antibacterial biomaterials from laboratory research to clinical applications also requires addressing challenges related to manufacturing and scalability. Techniques such as electrospinning and hydrogel fabrication are well established at the laboratory scale but may present difficulties when adapted to large-scale production. Parameters such as fiber morphology, polymer composition, and drug loading must be carefully controlled to ensure reproducibility and consistent material performance [61,103].

In addition, the integration of multiple components—including polymers, nanoparticles, and biological agents—can increase manufacturing complexity. Developing standardized fabrication protocols and scalable processing methods will therefore be essential for facilitating industrial production and regulatory approval.

### 5.4. Regulatory and Clinical Translation Challenges

Beyond technical considerations, regulatory and clinical challenges must also be addressed before multifunctional antibacterial biomaterials can be widely implemented in medical practice. Materials incorporating bacteriophages, antibiotics, and inorganic nanoparticles often fall within complex regulatory frameworks that require extensive safety and efficacy evaluation.

Importantly, not all antibacterial hydrogel strategies are equally advanced in terms of clinical readiness. Systems based on conventional antibiotics or inorganic components such as hydroxyapatite generally benefit from more established regulatory pathways and a clearer history of clinical use, particularly in bone-related and wound healing applications. In contrast, bacteriophage-loaded hydrogels remain at an earlier stage of development, largely due to challenges associated with phage stability, batch standardization, and regulatory classification as biological therapeutics. Similarly, electrospun and hybrid fiber–hydrogel systems, while highly promising from a materials design perspective, still face significant barriers related to scalable manufacturing, reproducibility, and integration into clinically approved fabrication processes. These differences highlight the need for a more nuanced and application-driven approach to the design of antibacterial hydrogel systems, in which material complexity is carefully balanced against translational feasibility and regulatory constraints.

Furthermore, regardless of their level of technological maturity, clinical validation of these systems requires well-designed studies that demonstrate clear advantages over existing treatments. Factors such as long-term biocompatibility, immune responses, and potential ecological impacts of bacteriophage use must be carefully evaluated before clinical adoption [104].

### 5.5. Future Research Directions

Despite these challenges, the future of antibacterial hydrogel systems appears highly promising. Continued advances in material science, nanotechnology, and biofabrication are likely to enable the development of increasingly sophisticated biomaterial platforms. In particular, the integration of biological antibacterial agents, inorganic functional components, and architectured polymer scaffolds offers exciting opportunities for creating multifunctional systems capable of simultaneously addressing infection control and tissue regeneration.

Within the tri-pillar framework proposed in this review, future research will likely focus on improving the synergistic interactions between these components, optimizing material architectures, and developing fabrication techniques that enable precise spatial organization of therapeutic elements. Such efforts may ultimately lead to next-generation antibacterial biomaterials capable of addressing some of the most pressing challenges associated with antibiotic-resistant infections and complex tissue repair. Emerging fabrication approaches such as additive manufacturing and 3D bioprinting are also being explored for the development of bacteriophage-loaded hydrogel systems with spatially controlled architectures [105].

Future studies should focus on quantitatively correlating material design parameters with antibacterial performance, developing standardized evaluation protocols, and advancing clinically relevant models to facilitate translation of multifunctional hydrogel systems into medical practice.

## 6. Conclusions

The development of antibacterial hydrogels represents a rapidly evolving field driven by the urgent need to address antimicrobial resistance and biofilm-associated infections. This review has examined recent advances through a conceptual framework based on the integration of biological, inorganic, and structural design strategies. The convergence of these three components enables the design of multifunctional hydrogel systems that simultaneously address infection control and tissue regeneration.

Bacteriophage-based approaches provide highly specific antibacterial activity and represent a promising alternative to conventional antibiotics, particularly for multidrug-resistant infections. Inorganic components such as hydroxyapatite contribute additional functionalities, including drug adsorption, modulation of the local microenvironment, and enhanced tissue integration. Structural design strategies based on electrospinning enable the fabrication of fibrous architectures that improve mechanical stability and regulate the delivery of therapeutic agents. The convergence of these three approaches allows for the design of hydrogel systems with enhanced functionality compared with single-component materials.

Despite these advances, several challenges remain. The stability and reproducibility of biological agents, particularly bacteriophages, require further optimization. In addition, the scalability of fabrication processes and the standardization of complex composite systems continue to limit clinical translation. Regulatory considerations also represent a significant barrier, especially for biologically active and multifunctional materials.

Future research should focus on the development of more robust and scalable fabrication strategies, improved control over the spatial and temporal release of therapeutic agents, and a deeper understanding of interactions between materials, bacteria, and host tissues. Recent research has focused on composite biomaterials integrating antibacterial agents, bioactive inorganic particles, and responsive polymer networks capable of adapting their behavior to environmental stimuli such as pH, enzymatic activity, temperature, or bacterial presence [106,107,108]. These stimuli-responsive systems represent a promising direction for achieving more precise and adaptive antibacterial therapies.

Overall, the convergence of biological, inorganic, and structural strategies provides a promising pathway for the development of advanced antibacterial hydrogels. By combining targeted antibacterial activity with controlled delivery and tissue regeneration capabilities, these systems have the potential to play a significant role in future therapeutic approaches for complex infections.

## Figures and Tables

**Table 1:** Comparative overview of the three design pillars in multifunctional antibacterial hydrogels, highlighting their primary functions, advantages, limitations, and contributions to overall system performance.

Pillar	Key Function	Advantages	Limitations	Contribution to Hydrogel Performance
Biological (Bacteriophages)	Targeted antibacterial activity and biofilm disruption	High specificity; self-amplification; effective against multidrug-resistant bacteria	Limited stability; sensitivity to environmental conditions; regulatory challenges	Enables selective bacterial eradication and reduces antibiotic resistance risks
Inorganic (Hydroxyapatite)	Drug adsorption and modulation of local microenvironment	High biocompatibility; osteoconductivity; tunable surface chemistry	Potential aggregation; limited intrinsic antibacterial activity; dependence on surface functionalization	Enhances drug loading capacity and supports tissue regeneration, especially in bone-related applications
Structural (Electrospinning)	Formation of fibrous architectures and controlled release	High surface area; tunable mechanical properties; ECM-mimicking structures	Scale-up challenges; potential solvent toxicity; heterogeneous drug distribution	Provides structural support, improves mechanical stability, and enables controlled release profiles

**Table 2:** Representative strategies for bacteriophage incorporation into hydrogel systems and associated design considerations.

Strategy	Hydrogel Type	Loading Method	Key Advantages	Main Limitations	Representative References
Direct entrapment	Natural & synthetic hydrogels	Mixing before gelation	Simple uniform distribution	Risk of inactivation during crosslinking	[8]
Diffusion loading	Preformed hydrogels	Soaking	Mild conditions	Burst release	[8,17]
Affinity-based systems	Functionalized systems	Electrostatic binding	Prolonged retention	Complex chemistry	[8]
Microencapsulation	Composite systems	Multi-step encapsulation	Enhanced stability	Manufacturing complexity	[17]
Stimuli-responsive systems	Smart hydrogels	Infection-triggered release	On-demand release	Limited clinical data	[8,29]
Hybrid fibrous-hydrogel systems	Electrospun composites	Layered architecture	Mechanical reinforcement	Fabrication complexity	[27,30]

**Table 3:** Representative hydroxyapatite-based hydrogel composites for antibacterial and bone-related applications.

HA Type	Hydrogel Matrix	Antibacterial Agent	Target Application	Key Outcome	References
Nano-HA	Gelatin/Alginate	Gentamicin	Osteomyelitis model	Sustained antibiotic release with improved antibacterial activity	[11,26]
Nano-HA (surface modified)	PEG-based hydrogel	Vancomycin	Bone defect infection	Reduced burst release and prolonged drug availability	[44]
Carbonated HA	Chitosan hydrogel	Ciprofloxacin	Bone tissue engineering	Enhanced adsorption capacity and controlled desorption kinetics	[43,45]
Nano HA	Hybrid hydrogel scaffold	Broad-spectrum antibiotics	Implant associated infection	Improved mechanical stability and local antibacterial effect	[10,46]

## Data Availability

No new data were created or analyzed in this study.
